# Clinical significance and simultaneous microevolution of two closely-related nontuberculous mycobacteria species

**DOI:** 10.3389/fmicb.2026.1791000

**Published:** 2026-04-07

**Authors:** Chengcheng Wang, Dan Zhou, Yu Feng, Feifei Zhao, Yuling Xiao, Yi Xie, Alan McNally, Zhiyong Zong

**Affiliations:** 1 Center of Infectious Diseases, West China Hospital, Sichuan University, Chengdu, China; 2 Division of Infectious Diseases, State Key Laboratory of Biotherapy, Chengdu, China; 3 Department of Laboratory Medicine, West China Hospital, Sichuan University, Chengdu, China; 4 Laboratory of Pathogen Research, West China Hospital, Sichuan University, Chengdu, China; 5 Institute of Microbiology and Infection, College of Medicine and Health, University of Birmingham, Birmingham, United Kingdom; 6 State Key Laboratory of Respiratory Health and Multimorbidity, Chengdu, China

**Keywords:** coinfection, microevolution, mycobacteria, mycobacterium septicum, *Mycolicibacterium nivoides*, non-tuberculosis mycobacteria

## Abstract

**Introduction:**

We aimed to investigate the clinical significance and in-host evolution of *Mycobacterium septicum* and *Mycolicibacterium nivoides*, two closely-related nontuberculous mycobacteria.

**Methods:**

Using a detailed single-case study, we investigated pulmonary co-infection of *M. septicum* and *M. nivoides* by examining clinical data, conducting microbiological assays, performing genome sequencing for 20 colonies from each sample, and evaluating virulence using *Galleria mellonella* larva. We conducted a literature review to summarize *M. septicum* and *M. nivoides* clinical cases and curated all publicly-accessible genomes.

**Results:**

In the pulmonary co-infection, both *M. septicum* and *M. nivoides* were implicated as the causative agents and exhibited remarkable intra-strain genomic heterogeneity. We uncovered different putative in-host microevolution trajectories, via either *α*/*β* hydrolase variation for *M. septicum* or *papA2* (trehalose-2-sulfate acyltransferase-encoding gene) mutation for *M. nivoides*. Intriguingly, both mutations were associated with enhanced virulence in the *G. mellonella* model. We identified 19 additional cases with *M. septicum* in literature, showing its multiple roles as a pathogen, a colonizer, or a contaminant. Re-analysis of 16 publicly-accessible genomes revealed misclassification of *M. nivoides*, potentially underestimating its clinical significance.

**Discussion:**

This study presents the first genomic evidence of co-infection of *M. septicum* and *M. nivoides* and divergent putative within-host evolution in a human patient. Simultaneously identifying multiple NTM colonies in chronic infection is needed for precise diagnosis. The observed convergence towards increased model virulence warrants further investigation as a potential adaptive strategy in NTM.

## Introduction

Non-tuberculous mycobacteria (NTM) are an increasingly recognized cause of pulmonary diseases, particularly in patients with underlying conditions or compromised immunity (Dahl et al., 2022). Among these, *Mycobacterium septicum*, first described in 2000 as the causative agent of a central line-associated bloodstream infection in a child with metastatic hepatoblastoma (Schinsky et al., 2000), has been implicated in various human infections (Adekambi and Drancourt, 2006; Hawkins et al., 2008; Miki et al., 2010; Lian et al., 2013; Go et al., 2020; Klomjit et al., 2020; Shin et al., 2020), though it remains relatively rare. *Mycolicibacterium nivoides* is a newly described species, first reported in 2021, and its clinical relevance is not yet well understood (Dahl et al., 2021). The high average nucleotide identity (ANI) (95.1%) between *M. septicum* DSM 44393^T^ and *M. nivoides* DL90^T^ suggests that they belong to a common species complex, further complicating accurate species-level identification. Incorrect assignation of *Mycobacterium* species has been well documented (Riojas et al., 2018; Meehan et al., 2021), although precise species identification lays the foundation for microbiological understanding and has crucial clinical implications (Parks et al., 2020; Zong, 2020). A few *M. septicum* and *M. nivoides* genomes are accessible in GenBank, which may need further careful curation for precise species assignation.

Of note, a study published in 2018 (Gupta et al., 2018) has transferred species of the “*Fortuitum-Vaccae*” clade from the genus *Mycobacterium* to a new genus named *Mycolicibacterium*. Subsequently, the new species names with *Mycolicibacterium* have been included in a validation list of International Journal of Systematic and Evolutionary Microbiology (Oren and Garrity, 2018), the official publication of the International Committee on Systematics of Prokaryotes and the Bacteriology and Applied Microbiology. However, it has also been proposed to keep using the original names with *Mycobacterium*, given that the new nomenclature has the potential to cause confusion and provides no benefits to the field of clinical mycobacteriology (Tortoli et al., 2019; Meehan et al., 2021). The original names with *Mycobacterium* remain validly published (Tortoli, 2019). We use the species names with *Mycobacterium* in this study and hereafter we apply *M.* for all *Mycobacterium* species names for brevity.

Cases of co-infections involving closely related NTM species have been reported but are uncommon and present unique diagnostic and clinical challenges (Conville et al., 1989; Naito et al., 2020). Such cases raise questions about potential interspecies interactions, shared pathogenicity mechanisms, and the host environment that facilitates their coexistence (Peters et al., 2012; Timme et al., 2024). Current diagnostic methods, such as matrix-assisted laser desorption/ionization-time-of-flight mass (MALDI-TOF MS) and 16S rRNA gene sequencing, often lack the resolution to distinguish closely related NTM species, leading to misidentification and underestimation of their clinical significance (Adékambi and Drancourt, 2004; Mediavilla-Gradolph et al., 2015). Whole genome sequencing (WGS) has emerged as a robust tool for precise identification and in-depth characterization, offering insights into genomic diversity and single nucleotide variations (SNVs), which are crucial for understanding NTM evolution and adaptation (Zhang et al., 2024).

We identified the rare and diagnostically challenging co-infection of *M. septicum* and *M. nivoides* in a case with persistent NTM pulmonary disease. By combining genomic, phenotypic, and clinical analyses, we characterized the distinct clonal backgrounds of the two species, elucidated their antimicrobial resistance profiles, and uncovered the genomic heterogeneity. Particularly, we revealed that the two species used different evolutional approaches toward enhanced virulence for long-term human adaptation. This case study aims to generate hypotheses about the clinical relevance and in-host evolution of these closely related species.

## Materials and methods

### Ethics statement

This study was approved by the Ethical Committee of West China Hospital (No. 20231206), and informed consent was obtained from the patient.

### Literature search strategy and selection criteria

We searched PubMed, Web of Science, Embase, Ovid MEDLINE, and Cochrane to identify studies including patients with *M. septicum* and *M. nivoides* published until August 25, 2025. The following terms were used: *Mycobacterium septicum* OR *Mycolicibacterium septicum* OR *M. septicum* OR *septicum; Mycobacterium nivoides* OR *Mycolicibacterium nivoides* OR *M. nivoides* OR *nivoides.* In addition, the reference lists of the retrieved studies were manually screened for additional literature. We included all kinds of studies and reports representing human infections caused by *M. septicum* and *M. nivoides*. We excluded studies that did not involve humans, such as wildlife or fish. We also excluded studies lacking clinical data.

### Isolate collection and purification

Isolates 120,309 and 120,310 were recovered from independent sputum samples collected from a patient as part of routine clinical care at West China Hospital, Chengdu, China, in 2023. The sputum specimens were obtained at two different clinical visits at about 1 month apart and were processed separately using standard mycobacterial culture procedures. Briefly, samples were decontaminated and inoculated into *Mycobacterium* Growth Indicator Tube (MGIT) liquid medium (BD, Franklin Lakes, NJ, USA) and onto Lowenstein-Jensen medium (BaSO, Zhuhai, China).

During the initial isolation of strains 120,309 and 120,310, multiple colonies were randomly collected from primary culture plates for whole-genome sequencing. We noticed a high “contamination” rate (86.1%) of the genome of isolate 120,309 as determined by CheckM v1.0.18 (Parks et al., 2015). As such, we went back to further purify isolate 120,309 by serially three-round plating a single colony and performed WGS for a purified single colony. The “contamination” rate determined by CheckM then reduced to 1.4%, suggesting that the single colony was purified. Therefore, we subsequently implemented a three-round purification procedure for each specimen, ultimately selecting single colonies for clonal amplification prior to whole-genome sequencing.

### Initial species identification

Initial species identification was performed using MALDI-TOF MS (Bruker Daltonics, Bremen, Germany) as previously described (Buckwalter et al., 2016). All measurements and data processing were carried out using the Microflex LT instrument (Bruker Daltonics) and BioTyper software version 3.1 (Bruker Daltonics). We followed the evaluation standard developed and verified by the manufacturer.

### Antimicrobial susceptibility testing

MICs of cefoxitin, amikacin, clarithromycin, azithromycin, rifabutin, moxifloxacin, gatifloxacin, linezolid, ethambutol, imipenem, tobramycin, rifampin, minocycline, doxycycline, and trimethoprim/sulfamethoxazole were determined according to the Clinical and Laboratory Standards Institute (CLSI) guidelines (CLSI, 2018), using the Drug Susceptibility Test Kit for Mycobacteria (Culture Method) (Encode, Zhuhai, China) for isolates 120,309, and 120,310.

### DNA extraction and whole-genome sequencing

For whole genome sequencing, genomic DNA of 120,309, 120,310, and their derivative colonies were manually prepared using the QIAamp DNA minikit (Qiagen; Hilden, Germany). After preparation of DNA sequencing libraries with the NEBNext Ultra II DNA Library Prep kit for Illumina (NEB; Ipswich, MA), WGS was performed on the Hiseq-10X platform (Illumina; San Diego, CA) with a paired-end 150-bp-layout. The generated short reads were trimmed using Trimmomatic v0.39 (Bolger et al., 2014) with default settings and then assembled into contigs using the SPAdes-based assembly pipeline Shovill v1.1.0^1^ with default settings.

For long-read sequencing, genomic DNA of 120,309_1 (the first *M. septicum* strain from the first sample 120,309) and 120,309_2 (the first *M. nivoides* strain from the first sample 120,309) were prepared with the Monarch genomic DNA purification kit (NEB) and was cleaned using AMPure XP beads (1:1) (Beckman Coulter; Brea, CA). Multiplexed libraries were prepared using the Rapid Barcoding Kit (Oxford Nanopore Technologies; Oxford, UK) and sequenced on a MinION system equipped with an R10.4.1 flow cell (Oxford Nanopore Technologies). Base calling and demultiplexing were conducted using Dorado v1.0.2^2^ with the config file dna_r10.4.1_e8.2_400bps_sup@v5.2.0. To generate multiple complete genome assemblies, long reads were input into four assemblers: Canu v2.2, Flye v2.9.1, miniasm v0.3_r179 (paired with minipolish v0.1.3), and Raven v1.8.1. Contigs from these different assemblies were clustered using Trycycler v0.5.5, with a consensus contig generated for each cluster. These consensus contigs underwent sequential polishing using Medaka v2.1.0^3^, Polypolish v0.5.0, and pypolca v0.3.1^4^ to produce the final high-quality assembly. All software was run with default parameters unless stated otherwise.

### Strain typing and gene prediction

A quality check was performed using CheckM v1.0.18 (Parks et al., 2015) to determine genome completeness and contamination. Genomes that passed this quality check were annotated using Prokka v1.14.5 (Seemann, 2014). Antimicrobial resistance genes were predicted using AMRFinderPlus v3.9 (Feldgarden et al., 2019).

### Precise species identification and clonal relatedness determination

ANI between isolates 120,309, 120,310 and type strains of *Mycobacterium* species within the “*Fortuitum-Vaccae*” clade were determined using the fastANI v1.32 (Jain et al., 2018) with the recommended parameters and default settings, respectively. A ≥ 95% ANI (Richter and Rossello-Mora, 2009; Ciufo et al., 2018) was used as the cutoff to define a bacterial species.

All accessible genome sequences labeled *M. septicum* and *M. nivoides* in GenBank (accessed by 15-07-2025) were retrieved. PCR amplicon libraries were discarded. Raw sequencing reads were assembled into contigs by the SPAdes-based assembly pipeline Shovill v1.1.0 (see text footnote 1). For those that could not be assembled by Shovill v1.1.0, Velvet (Zerbino et al., 2009) was attempted. Genomes were subjected to quality check using CheckM v1.0.18 (Parks et al., 2015) and those in low quality, defined by >300 contigs, <90% genome completeness for individual genomes, were discarded.

### Single nucleotide variants (SNV) calling and predicted function

The complete genomes of *M. septicum* colony 120,309_1 and *M. nivoides* colony 120,309_2 were used as reference genomes for mapping. Snippy v4.6.0^5^ with default settings was used to map each colony against the selected references to identify SNVs. To assess the potential functional implications of the observed SNVs, we focused on nonsynonymous variations. These variations were further analyzed for functional predictions using tools including Uniprot,^6^ eggNOG-mapper,^7^ KEGG,^8^ Prokka v1.14.5 (Seemann, 2014), and NCBI Prokaryotic Genome Annotation Pipeline.^9^

### Clonal relatedness determination of *M. septicum* and *M. Nivoides* from the two samples

Phylogenomic trees of the 22 *M. nivoides* colonies and 18 *M. septicum* colonies from the two samples were inferred using *M. septicum* colony 120,309_1 and *M. nivoides* colony 120,309_2 as references, separately. Snippy v4.6.0 (see text footnote 5) with default settings was used to map each colony against the selected reference to identify SNVs. An alignment of sequences was generated by applying SNVs of each colony onto the reference genomes, followed by recombination filtering using Gubbins v2.4.1 (Croucher et al., 2015), with a maximum of 50 iterations under the GTR-GAMMA model. The recombination-free maximum-likelihood tree was then inferred using RAxML v8.2.12 (Stamatakis, 2014) under the GTR-GAMMA model with 1,000 iterations of bootstrapping. Trees were viewed and annotated using iTOL v6.9 (Letunic and Bork, 2021).

### Phylogenomic analysis of the *Mycobacterium* “*Fortuitum-Vaccae*” clade

To confirm the taxonomic relationship between *M. septicum* and *M. nivoides* and validate their status as distinct species, a phylogenomic analysis based on the concatenated protein sequences encoded by marker genes of type or reference strains of all species within the “*Fortuitum-Vaccae*” clade of *Mycobacterium* (Supplementary Table S1) was inferred using IQ-TREE v2.3.0 (Minh et al., 2020) under LG model allowing for sites heterogeneity with 1,000 ultra-fast bootstraps, and was visualized and annotated using iTOL v6.9 (Letunic and Bork, 2021). This analysis aimed to contextualize the two species within the broader genus and support the proposal of the ‘*M. septicum-nivoides* complex’ for closely related strains that are indistinguishable by conventional methods.

### Virulence assays

*Galleria mellonella* larva were used as an established *in vivo* model for evaluating NTM virulence, as their innate immune system (hemocytes) mimics mammalian phagocytic responses (neutrophils and macrophages) and survival correlates with clinical pathogenicity in human infections (Meir et al., 2018; Pereira et al., 2018; Asai et al., 2019). This model has been validated for assessing virulence of rapidly growing mycobacteria (e.g., *M. abscessus*) and provides a robust, ethical alternative to mammalian models for initial functional characterization of mutations (Meir et al., 2018; Asai et al., 2019).

Virulence assays were performed using *G. mellonella* larva, weighing ∼180 to 200 mg (Huiyude Biotech, Tianjin, China) for four strains of two pairs. One pair was strains 120,309_1 (the first *M. septicum* colony from the first sample 120,309) and 120,310_9 (from sample 120,310), characterized by the Ile175Val substitution in the *α*/*β* hydrolase gene and the absence of any other SNVs compared to 120,309_1. Another pair was the strains 120,309_2 (the first *M. nivoides* colony from sample 120,309) and 120,310_11 (from sample 120,310), characterized by a nucleotide deletion in the *papA2* gene and the absence of any other SNVs compared to 120,309_2. For each group, 16 larvae per group were injected with 10 μL suspension containing increasing bacterial doses (from 7.5 × 10^8^, 1.5 × 10^9^, 5 × 10^9^, 2.5 × 10^10^, to 5 × 10^10^ CFU/mL) of 120,309_1 and 120,310_9, 120,309_2 and 120,310_11, or 10 μL PBS as a negative control (Supplementary Tables S2, S3), with significant inter-group differences occurring at 10 μL of 5 × 10^9^ CFU/mL (Supplementary Tables S4, S5). The assays were performed in triplicate, which were pooled (48 larvae per group) for statistical analysis using Log-rank test.

## Results

### *M. septicum* causing pulmonary infection in an elderly patient

A 74-year-old male patient was admitted to a local hospital in June 2023, due to two-week fever (highest axillary temperature, 38 °C), cough, sputum, and sore throat, accompanied by muscle soreness and dizziness. He had prostate cancer for 8 years with continuously taking goserelin acetate (Zoladex depot, 3.6 mg per 4 weeks) and xbicalutamide (150 mg/d). A chest computer tomography (CT) on admission to that hospital showed slight inflammation and a few small nodular shadows (0.2–0.5 cm in size) scattered in both lungs as well as bilateral pleural thickening (Supplementary Figure S1). He received penicillin G for 7 days at the local hospital but had no response. Therefore, he came to our outpatient clinic for consultation.

On the day of this outpatient visit, routine blood tests in the outpatient showed an elevated white blood cell count (12.78 × 10^9^/L) with 79.9% neutrophils and 14.0% lymphocytes. Biochemistry, myocardial enzymes, thyroid function, and anti-streptolysin O were normal. Tests for Epstein–Barr virus nucleic acids and IgM of toxoplasmosis, rubella, cytomegalovirus, and herpes simplex virus were negative. Sputum samples were collected on the day of this outpatient visit for conventional bacterial culture, acid-fast smears, and mycobacterial culture. No acid-fast bacilli were detected in smears, and no bacteria grew from cultures on blood agar at 35 °C for 48 h although the positive control, *Streptococcus pneumoniae* ATCC 49619, grew. He was empirically administered oral azithromycin (0.5 g, qd) for 3 days, after which fever, cough, sore throat, muscle soreness, and dizziness resolved, but mild cough with scant sputum persisted. One week after his outpatient visit, the mycobacterial culture of sputum grew bacteria (isolate 120309) in *Mycobacterium* liquid medium (BD, Franklin Lake, NJ) and then on Lowenstein-Jensen medium (BaSO, Zhuhai, China), which was identified as *M. septicum* by MALDI-TOF MS later. Two months after his outpatient visit, the patient reported no improvement in cough or sputum, but no constitutional symptoms (e.g., weight loss and night sweats). A follow-up chest CT scan 1 month later showed that the slight inflammation and small nodules still existed. The patient’s sputum was collected for mycobacterial culture again, which also grew mycobacteria (isolate 120310) and was subsequently identified as *M. septicum* by MALDI-TOF MS. This case had two sputum cultures positive for NTM, met the clinical (pulmonary symptoms) and radiologic requirements, and therefore fulfill the criteria for diagnosis of NTM pulmonary diseases (Daley et al., 2020). *In vitro* susceptibility was tested to determine minimum inhibitory concentrations (MIC) according to CLSI guidelines (CLSI, 2018). Both isolates exhibited similar antimicrobial resistance profiles with identical susceptibility categories and MICs of 15 antimicrobial agents were shown in Supplementary Table S6.

Differential diagnosis revealed no infections other than *M. septicum*. While metastatic prostate cancer lesions could not be definitively excluded due to his oncologic history, the stable respiratory symptoms, absence of signs of metastasis suggested a more indolent infection than aggressive malignancy. A multidrug regimen (amikacin, tigecycline, cefoxitin, and moxifloxacin) was proposed, but the patient declined treatment due to concerns about side effects, and reluctance to undergo medical intervention.

### The co-existence of isolates belonging to two closely related species, *M. septicum* and *M. Nivoides*

Due to the rare identification of *M. septicum* from clinical samples and the limited resolution of MALDI-TOF MS for identifying NTM (52.3–98.4%) (Mediavilla-Gradolph et al., 2015; Quinlan et al., 2015; Rindi et al., 2022), we obtained the draft genome sequences of the two isolates by WGS and then performed ANI analyses to achieve precise species identification. Unexpectedly, the two isolates exhibited discordance in ANI analysis. Isolate 120,309, the first isolate had the highest ANI value (99.0%) with the type strain of *M. nivoides* (DL90^T^, accession no. CP034072) and a lower ANI (94.9%) with *M. septicum* DSM 44393^T^ (accession no. CBMO000000000). In contrast, isolate 120,310 had the highest ANI (95.7%) with *M. septicum* DSM 44393^T^ and a lower ANI (94.6%) with *M. nivoides* DL90^T^. This suggests that the two isolates could belong to two different, yet-closely-related, species. We therefore performed careful examination of their genome sequences and noticed a high “contamination” rate (86.1%) of the genome of isolate 120,309 in contrast to only 0.4% of the other isolate as determined by CheckM v1.0.18 (Parks et al., 2015). As such, we went back to further purify isolate 120,309 by serially three-round plating a single colony and performed WGS for a purified single colony. The “contamination” rate determined by CheckM then reduced to 1.4%, suggesting that the single colony was purified. Nevertheless, the genome sequence of this purified isolate 120,309 still had a 99.1% ANI with *M. nivoides* DL90^T^ and a lower ANI (94.9%) with *M. septicum* DSM 44393^T^, similar to that before purification. In addition, the ANI between isolates 120,309 and 120,310 is 94.6%. The above findings suggest that isolates 120,309 and 120,310 belonged to two different species.

**Figure 1:**
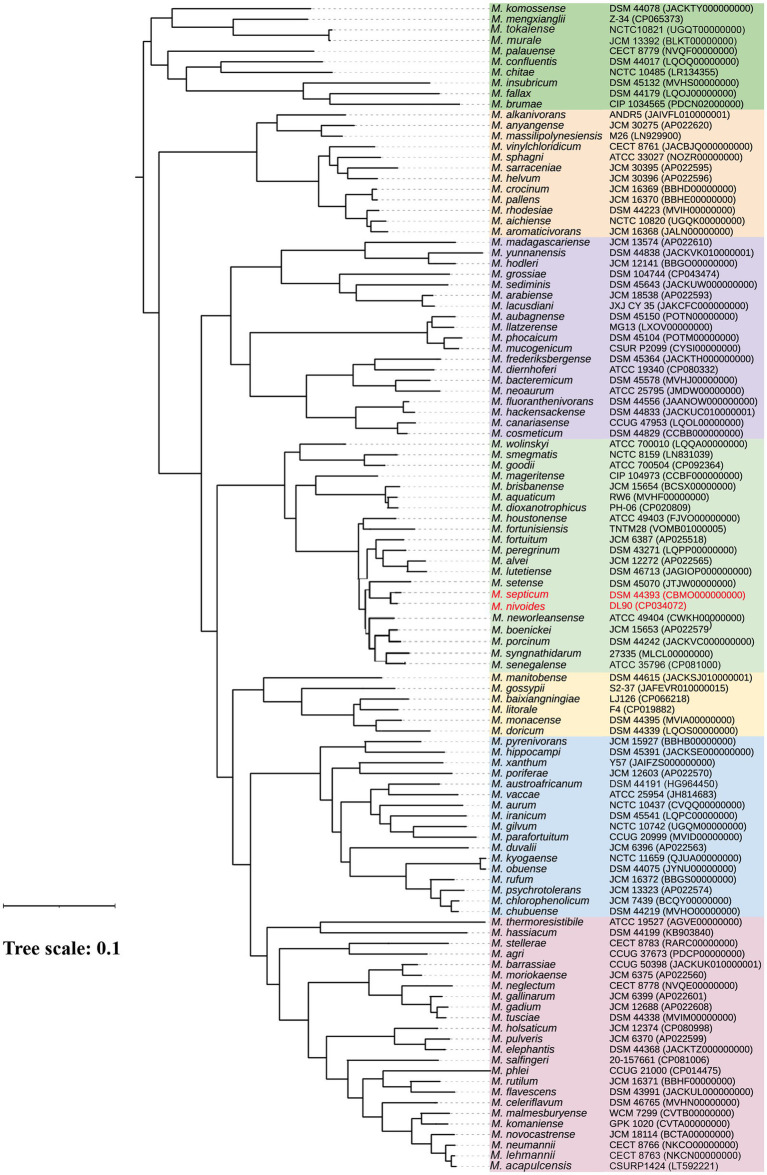
A phylogenomic tree of species within the *Mycobacterium* “*Fortuitum-Vaccae”* clade. The tree was inferred based on the concatenated nucleotide sequence of the core genes of type or reference strains of species within the *M. fortuitum-Vaccae* clade (listed in Supplementary Table S1). Strains and their nucleotide accession numbers are listed alongside the species names. Bar, value indicates the nucleotide substitutions per site. *M. septicum* is closely related to *M. nivoides* (ANI, 95.2%) and *M. setense* (ANI, 88.1%) within the major branch containing the well-characterized *M. fortuitum*.

The subsequential detection of two closely related *Mycobacterium* species from the same infection site of a single patient within a two-month period is intriguing. We therefore investigated the clonal relatedness and the taxonomic position of *M. septicum* and *M. nivoides*. We performed a phylogenomic analysis of all type or reference strains of species within the “*Fortuitum-Vaccae*” clade of *Mycobacterium*. The inferred phylogenomic tree (Figure 1) illustrates that *M. septicum* cluster together with *M. nivoides* and *M. setense* within a large branch containing the well-characterized *M. fortuitum*. This branch also includes *M. alvei*, *M. boenickei*, *M. fortunisiensis*, *M. houstonense*, *M. lutetiensei, M. neworleansense*, *M. peregrinum*, *M. porcinum*, *M. senegalense*, *M. setense*, and *M. syngnathidarum. M. septicum* and *M. nivoides* have a > 95% ANI, whereas they share <93% ANI with other species, up to 88.1% ANI with *M. setense* (Supplementary Table S7), their closest relative species. This suggests that *M. septicum* and *M. nivoides* are indeed closely related and form a common species complex, for which we assigned the *M. septicum-nivoides* complex thereafter.

### *M. septicum* and *M. Nivoides* in the patient are heterogenous in clonal background

Although isolates belonging to different species were subsequentially identified from two samples, it is possible that the patient was simultaneously infected by isolates of both species rather than a single isolate. This is supported by the high “contamination” rate of (86.1%) of the genome of isolate 120,309 as heterogeneity can be regarded as “contamination” by CheckM (Parks et al., 2015). To test this hypothesis, we randomly selected 20 single colonies from each of the two isolates on the original plate, serially plated each single colony in three rounds, and obtained the draft genome sequence of all 40 purified colonies by WGS. For isolate 120,309, we found that 15 of the 20 single colonies belonged to *M. nivoides* as they had a 99.08 to 99.11% ANI with DL90^T^ but a lower one (94.94 to 95.04%) with *M. septicum* DSM 44393^T^ (Table 1). Conversely, the remaining five were likely of *M. septicum* (sharing a 95.75 to 95.77% ANI with DSM 44393^T^) rather than *M. nivoides* (94.61 to 94.69% with DL90^T^). In contrast, for isolate 120,310, 13 colonies were likely of *M. septicum* (95.72 to 95.79% ANI with DSM 44393^T^) rather than *M. nivoides* (94.63 to 94.72% with DL90^T^), whereas the other seven belonged to *M. nivoides* (99.08 to 99.10% ANI with DL90^T^ vs. 94.93 to 95.04% ANI with *M. septicum* DSM 44393^T^). This indicates that the patient was simultaneously infected by both *M. septicum* and *M. nivoides*.

**Table 1:** ANI analysis between 120,309 and 120,310 derived isolates and type strains of *M. septicum* and *M. nivoides.*

Isolate name	*M. nivoides* DL90^T^	*M. septicum* DSM 44393^T^	Contamination rate
120,309_1	94.7	95.7	0.4
**120,309_2**	99.1	94.9	1.5
**120,309_3**	99.1	95.0	1.5
**120,309_4**	99.1	95.0	1.5
120,309_5	94.7	95.8	0.4
**120,309_6**	99.1	95.0	1.5
**120,309_7**	99.1	95.0	1.5
120,309_8	94.6	95.8	0.4
**120,309_9**	99.1	95.0	1.5
**120,309_10**	99.1	95.0	1.5
**120,309_11**	99.1	95.0	1.5
120,309_12	94.7	95.8	0.4
**120,309_13**	99.1	95.0	1.5
120,309_14	94.6	95.8	0.4
**120,309_15**	99.1	95.0	1.5
**120,309_16**	99.1	94.9	1.5
**120,309_17**	99.1	95.0	1.5
**120,309_18**	99.1	95.0	1.5
**120,309_19**	99.1	95.0	1.5
**120,309_20**	99.1	95.0	1.5
**120,310_1**	99.1	95.0	1.5
120,310_2	94.7	95.7	0.4
120,310_3	94.7	95.8	0.4
120,310_4	94.6	95.7	0.4
120,310_5	94.7	95.8	0.4
120,310_6	94.7	95.8	0.4
**120,310_7**	99.1	95.0	1.5
120,310_8	94.6	95.7	0.4
120,310_9	94.7	95.8	0.4
120,310_10	94.7	95.8	0.4
**120,310_11**	99.1	95.0	1.5
**120,310_12**	99.1	94.9	1.5
120,310_13	94.7	95.8	0.4
**120,310_14**	99.1	95.0	1.5
**120,310_15**	99.1	94.9	1.5
120,310_16	94.7	95.8	0.4
**120,310_17**	99.1	95.0	1.2
120,310_18	94.7	95.8	0.4
120,310_19	94.7	95.8	0.4
120,310_20	94.6	95.8	0.4

Those belonged to *M. nivoides* are highlighted in bold.

**Figure 2:**
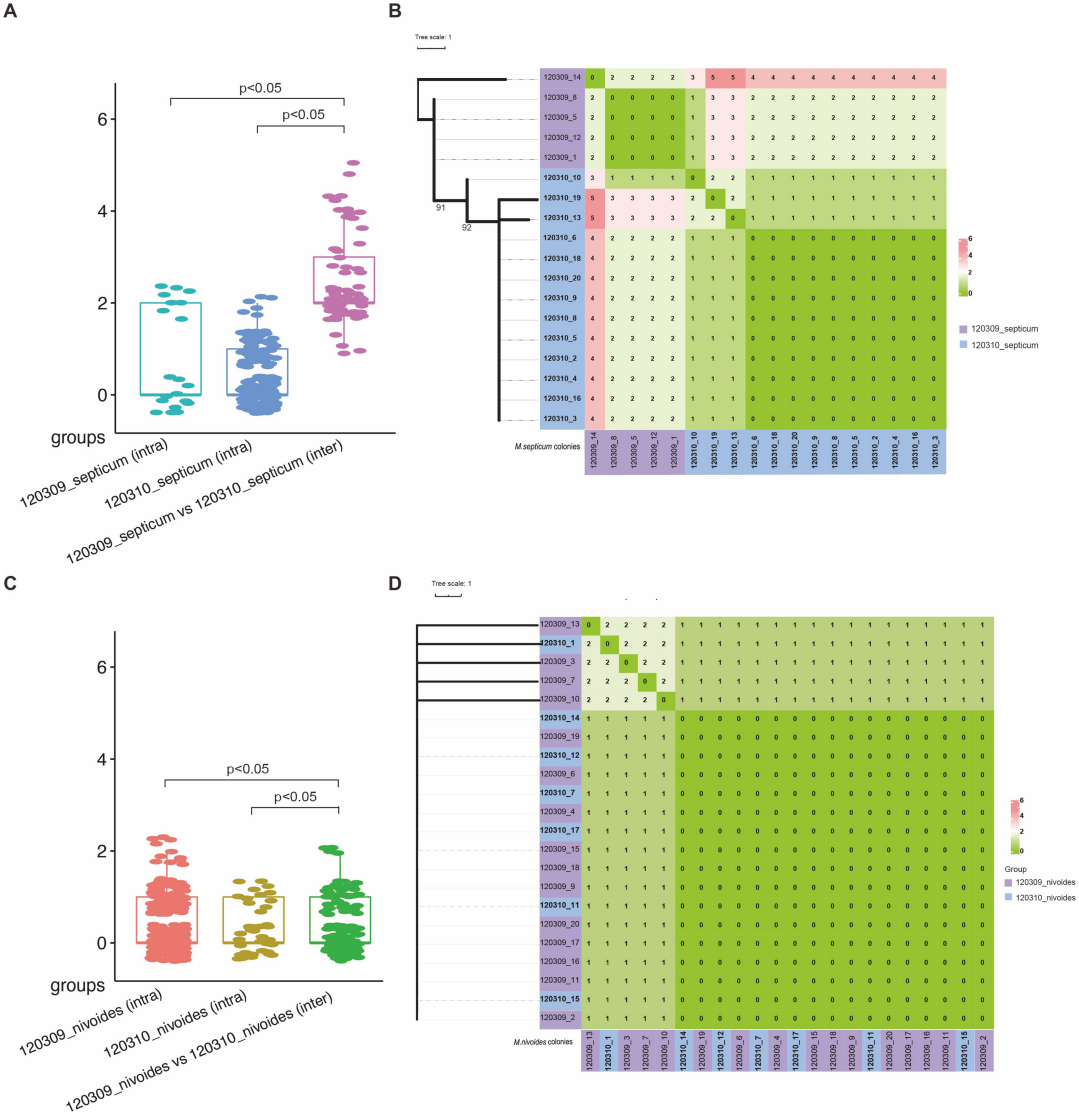
Clonal relatedness determination of *M. septicum* and *M. nivoides* from the two samples of the patient. **(A)** Boxplot of pairwise SNVs of intra-group and inter-group of *M. septicum* colonies. **(B)** Phylogenomic tree and heat map showing clustering of 18 *M. septicum* colonies based on pairwise SNVs. The tree was inferred using 120,309_1 (*M. septicum*) as the reference. **(C)** Boxplot of pairwise SNVs of intra-group and inter-group of *M. nivoides* colonies. **(D)** Phylogenomic tree and heat map showing clustering of 22 *M. nivoides* colonies based on pairwise SNVs. The tree was inferred using 120,309_2 (*M. nivoides*) as the reference. Trees were midpoint rooted with bootstrap support over 50% shown in gradients. SNVs were identified via Snippy v4.6.0, aligned to the reference genomes, and filtered for recombination using Gubbins v2.4.1 under the GTR-GAMMA model. Recombination-free phylogenies were inferred with RAxML v8.2.12 using 1,000 bootstrap iterations. Trees were visualized and annotated with iTOL v6.9.

To gain further insights into the genomic diversity of the *M. septicum-nivoides* complex in the patient, we called pairwise SNVs using Snippy (see text footnote 5). Among the 20 colonies recovered from the first sputum sample (corresponding to isolate 120,309), the 15 *M. nivoides* colonies had 0–2 pairwise SNVs, and the five *M. septicum* ones also processed 0–2 SNVs (Figure 2 and Supplementary Dataset S1). In contrast, there were 196,211–197,208 pairwise SNVs between the 15 *M. nivoides* and the five *M. septicum* colonies. Similarly, among the 20 colonies recovered from the second sputum sample (corresponding to isolate 120,310), seven *M. nivoides* and 13 *M. septicum* colonies had 0–1 and 0–2 pairwise SNVs, respectively, but had 196,450–198,521 SNVs between each other. We also determined pairwise SNVs between colonies recovered from different samples. The inter-sample pairwise SNVs of all *M. nivoides* colonies (*n* = 22) and all *M. septicum* ones (*n* = 18) from the two sputum samples were 0–2 and 1–5, respectively.

In the maximum-likelihood phylogenomic tree of *M. septicum*, the 18 colonies from the two samples were clustered separately (Figure 2). There were five nucleotide variations comprising one synonymous and four nonsynonymous mutations among the 18 *M. septicum* colonies (Table 2, Supplementary Figure S2, and Supplementary Dataset S2). To understand the potential functional implications of the observed SNVs, we examined and focused on nonsynonymous variations. Three nonsynonymous mutations led to amino acid substitutions of the cytochrome P450, a regulator of polyketide synthase expression, and a uroporphyrinogen-III methyltransferase/synthase in three different colonies (one mutation per colony), respectively (Table 2). Notably, the remaining nonsynonymous mutation resulted in an amino acid substitution (Ile175Val) of an *α*/*β* fold hydrolase and was present in all 13 colonies recovered from the second sample but was absent from the five from the first sample.

**Table 2:** Detection and functional prediction of nonsynonymous mutations in *M. septicum-nivoides* colonies.

Gene^a^	Nt mutation	Aa substitution	Predicted function	No. colonies
*M. septicum* (*n* = 18)				
ACLDT4_11630	A523G	Ile175Val	α/β hydrolase/putative aminoacrylate hydrolase RutD	13
ACLDT4_12570	G547T	Ala183Ser	Cytochrome p450	1
ACLDT4_09155	G1010A	Arg337His	Siroheme synthase/uroporphyrinogen-III synthase	1
ACLDT4_24405	A1048G	Ile350Val	PucR family transcriptional regulator/Regulator of polyketide synthase expression	1
*M. nivoides* (*n* = 22)				
ACLRC6_05035	G529del	Ala177fs	Trehalose-2-sulfate acyltransferase papA2/condensation domain Containing protein	4
ACLRC6_15640	G157del	Ala53fs	NADPH-dependent stearoyl-CoA 9-desaturase/fatty acid desaturase family protein	2
ACLRC6_08130	A631G	Thr211Ala	Short-chain-enoyl-CoA hydratase	1
ACLRC6_27725	T737G	Val246Gly	galE; UDP-glucose 4-epimerase	1

a Gene names such as ACLDT4_1163 and ACLRC6_05035 are generated through the automated annotation process of the NCBI Prokaryotic Genome Annotation Pipeline, originating from *M. septicum* colony 120,309_1 (GenBank accession no. JBKFEK000000000) and *M. nivoides* colony 120,309_2 (GenBank accession no. JBKQXC000000000), respectively.

aa, amino acid; del, deletion; fs, frameshift mutation; nt, nucleotide; Ile, Isoleucine; Val, Valine; Ala, Alanine; Ser, Serine; Arg, Arginine; His, Histidine; Thr, Threonine; Gly, Glycine.

Unlike *M. septicum*, the 22 *M. nivoides* colonies from the two samples were overlapped in the maximum-likelihood phylogenomic tree (Figure 2). A total of six nucleotide variations comprising two synonymous and four nonsynonymous ones were found in the *M. nivoides* colonies (Supplementary Dataset S2). Among the four nonsynonymous variations, only one was present in more than two colonies, specifically in four colonies from the second sputum sample (Table 2 and Supplementary Figure S2). It was a deletion of a nucleotide resulting in frameshift of *papA2* (Table 2), which encodes a trehalose-2-sulfate acyltransferase.

### Mutations in α/β fold hydrolase and *papA2* enhance virulence of *M. septicum* and *M. Nivoides*

Given that the α/β fold hydrolase mutation (Ile175Val) was exclusively detected in colonies from the second sample and the *papA2* frameshift mutation (from a single nucleotide deletion) was identified in four colonies of the same second sample. This suggests potential associations between these mutations and phenotypic changes. We further validated their impacts on bacterial virulence using *Galleria mellonella* infection assays, comparing the virulence of parental strains (120,309_1 for *M. septicum*, 120,309_2 for *M. nivoides*) with their respective mutant strains (120,310_9 carrying the α/β fold hydrolase mutation, 120,310_11 carrying the *papA2* frameshift mutation, both free of other SNVs).

**Figure 3:**
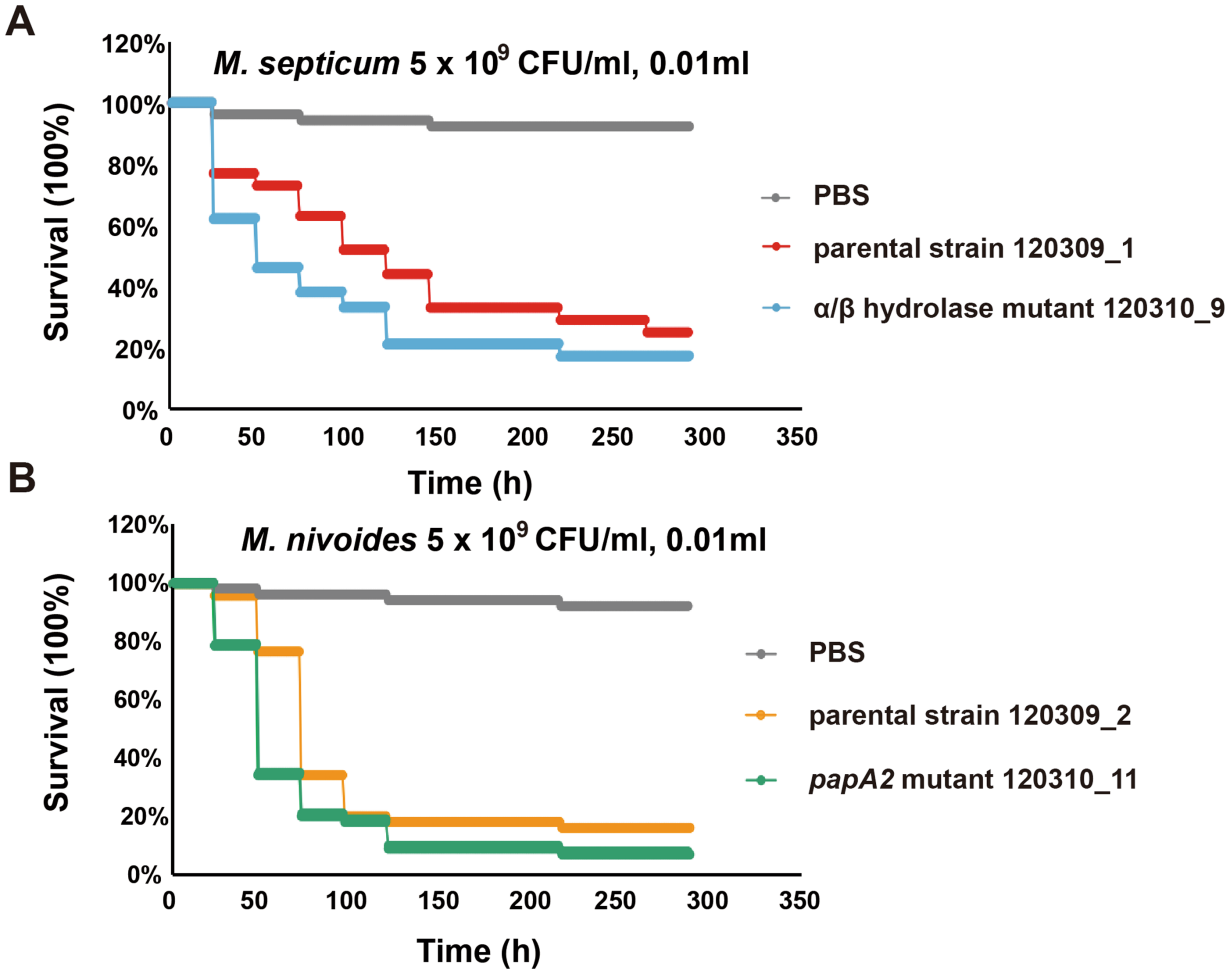
**(A)** Survival curves of *G. mellonella* larvae infected with the parental *M. septicum* strain 120309_1 and *α*/*β* hydrolase mutant 120310_9. **(B)** Survival curves of *G. mellonella* larvae infected with the parental *M. nivoides* strain 120309_2 and *papA2* mutant 120310_11.

Results revealed that the α/β fold hydrolase mutation markedly enhanced *M. septicum* virulence: larvae infected with mutant 120,310_9 had a survival rate of 45.83%, which was significantly lower than the 72.92% survival rate of larvae infected with parental strain 120,309_1 (*p* < 0.05; Figure 3). Similarly, the *papA2* frameshift mutation boosted *M. nivoides* virulence: at 48 h post-infection, larvae infected with mutant 120,310_11 showed a survival rate of 35.42%, significantly lower than the 77.08% survival rate observed with parental strain 120,309_2 (p < 0.05; Figure 3). No significant mortality was observed in the PBS control group, confirming that larval death was attributable to bacterial infection rather than experimental manipulation.

### Clinical significance of the *M. septicum-nivoides* complex as a pathogen, a colonizer, or a contaminant

To understand the clinical significance of the *M. septicum-nivoides* complex, we performed a literature review and identified 19 additional cases with *M. septicum* but no case with *M. nivoides* in the literature (Table 3) (Schinsky et al., 2000; Adekambi and Drancourt, 2006; Hawkins et al., 2008; Miki et al., 2010; Lian et al., 2013; Go et al., 2020; Klomjit et al., 2020; Shin et al., 2020). This narrative review, while summarizing the available case reports to contextualize our findings, was not conducted as a formal systematic review. Therefore, it does not support definitive quantitative conclusions about pathogenicity rates. Its purpose is to illustrate the spectrum of clinical presentations associated with *M. septicum-nivoides* complex and to highlight the historical diagnostic challenges within this complex. Notably, species identification was achieved by MALDI-TOF MS (*n* = 12) (Go et al., 2020) sequencing housekeeping genes (*n* = 5) comprising 16S rRNA alone (*n* = 3) (Schinsky et al., 2000; Adekambi and Drancourt, 2006; Hawkins et al., 2008), *hsp65* and *rpoB* (*n* = 1) (Lian et al., 2013), and the three genes in combination (*n* = 1) (Miki et al., 2010), or polymerase chain reaction (PCR) (without specifying the target gene, *n* = 1) (Shin et al., 2020), while the remaining one (Klomjit et al., 2020) report did not specify the method. It is worth to point out that both MALDI-TOF MS and housekeeping gene sequencing lacks the resolution to reliably distinguish closely related *Mycobacterium* species (Adékambi and Drancourt, 2004; Mediavilla-Gradolph et al., 2015; Quinlan et al., 2015; Rindi et al., 2022), possibly leading to misidentification of *M. nivoides* in clinical practice.

**Table 3:** An overview of clinical characteristics, therapy provided, and outcomes of patients with *M. septicum* in literature.

Case	Age/Sex	Comorbidity (s)	Sample	Catheter type	Immuno-suppression	Pathogen or not	Therapy (duration)^c^	Outcome	References
1	74/M	Prostate cancer	Sputum	/	Yes	Pathogen	None	Survived	This study
2	54/M	Systemic sclerosis, ESKD^a^	Ascites	PD	No	Pathogen	Catheter removal; LZD + MXF (4 m)	Death	Go et al. (2020)
3	77/F	Bronchiectasis, asthma	Sputum	/	No	Possible pathogen	MXF + R + CLR + AMK (15 m); MXF + R + CFZ + AMK (3 m); MXF + R + CFZ (4 y)	Survived	Go et al. (2020)
4	73/M	Bronchiectasis	Sputum	/	No	Colonizer	None	Survived	Go et al. (2020)
5	76/F	Tongue squamous cell cancer	Lymph node	/	No	Contaminant	Aspiration; none	Survived	Go et al. (2020)
6	75/M	Rheumatoid arthritis	Sputum	/	Yes	Colonizer	None	Survived	Go et al. (2020)
7	48/M	Cystic fibrosis	Sputum	/	No	Colonizer	None	Survived	Go et al. (2020)
8	75/F	Bronchiectasis, Crohn’s disease	Sputum	/	No	Possible pathogen	None (refused treatment)	Survived	Go et al. (2020)
9	67/F	Bronchiectasis	Sputum	/	No	Colonizer	None	Survived	Go et al. (2020)
10	54/M	None	Leg tissue	/	No	Contaminant	Transtibial amputation; none	Survived	Go et al. (2020)
11	67/F	Bicuspid aortic valve s/p AVR^a^	Shoulder tissue	/	No	Contaminant	None	Survived	Go et al. (2020)
12	57/M	None	Calf tissue	/	No	Contaminant	Irrigation and debridement; none	Survived	Go et al. (2020)
13	80/M	Rheumatoid arthritis, bronchiectasis	Sputum	/	No	Colonizer	None	Survived	Go et al. (2020)
14	53/M	ESKD^a^ and PH^a^ due to SS^a^	Ascites	PD^b^	Yes	Pathogen	Catheter removal; MXF + DOX (4 m)	Death	Klomjit et al. (2020)
15	37/F	Postblepharoplasty	Orbital tissue	/	No	Pathogen	LVF + CLR (6 m); the mass excised	Survived	Shin et al. (2020)
16	42/M	/	Sputum	/	No	Pathogen	HRZE (2 m)/HR (4 m), LVF (1 m)	Improved, relapsed 1 y later	Lian et al. (2013)
17	62/F	Schizophrenia	Sputum	/	No	Pathogen	CLR + LVF (2 m)	Survived	Miki et al. (2010)
18	47/M	Gut dysmotility	Blood	CVC^b^	No	Pathogen	TMP/SMX + AMP (NA)	Survived	Hawkins et al. (2008)
19	78/F	Hypothyroidism and hypertension	Sputum	/	No	Possible pathogen	CRO (10 d)	Improved, relapsed 2 y later	Adekambi and Drancourt (2006)
20	2/M	Metastatic hepatoblastoma	Blood, CVC^b^ tip	CVC^b^	Yes	pathogen	CVC^b^ removal; TMP/SMX (NA)	Survived	Schinsky et al. (2000)

a ESKD, end-stage of kidney disease; AVR, aortic valve replacement; PH, portal hypertension; SS, systemic sclerosis.

b CVC, central venous catheter; PD, Peritoneal dialysis.

c SCF, cefoperazone sulbactam; LVF, levofloxacin; MXF, moxifloxacin; LZD, linezolid; CLR, clarithromycin; AMK, amikacin; CFZ, clofazimine; DOX, doxycycline; H, isoniazid; R, rifampin; Z, pyrazinamide; E, ethambutol; TMP/SMX, trimethoprim-sulfamethoxazole; AMP, Ampicillin; CRO, ceftriaxone; d, day; m, month; y, years; NA, not available. None, no antimicrobial therapy.

Based on the judgment claimed in the corresponding reports, *M. septicum* was regarded as a pathogen for pneumonia (n = 2) (Miki et al., 2010; Lian et al., 2013), bloodstream infections (*n* = 2) (Schinsky et al., 2000; Hawkins et al., 2008), peritonitis (*n* = 2) (Go et al., 2020; Klomjit et al., 2020), and eye infection (*n* = 1) (Shin et al., 2020) in seven cases and as a possible pathogen for pneumonia in three other cases (Adekambi and Drancourt, 2006; Go et al., 2020). For the remaining cases, *M. septicum* was claimed as a colonizer (*n* = 5) (Go et al., 2020) or a contaminant (*n* = 4) (Go et al., 2020). Among the 10 cases that *M. septicum* was claimed as a pathogen or a possible pathogen, one refused treatment and survived, while 9 received antimicrobial therapy with varied agents and duration. Of the 9 patients, six survived after treatment, two improved but relapsed 1–2 years later with unknown final outcome due to the absence of data, and two died.

### Precise species identification of the *M. septicum-nivoides* complex in GenBank

As abovementioned, no publication reporting human infection due to the *M. septicum-nivoides* complex used WGS for precise species identification, it is likely that *M. nivoides* in clinical cases have been overlooked. We therefore sought to mine publicly available genomes of isolates recovered from clinical specimens to verify the species identification with linking literature and genomic data. This may allow us to identity additional species associated with human infection.

We examined and retrieved all accessible genome sequences labeled *M. septicum* (*n* = 25; comprising six genome assemblies and 19 raw reads) and *M. nivoides* (*n* = 4; comprising two genome assemblies and 2 raw reads) from GenBank (accessed by July 15, 2025). We deleted PCR amplicon libraries (*n* = 3) and raw sequencing reads (*n* = 3), which were unable to be assembled into contigs by the SPAdes-based assembly pipeline Shovill v1.1.0 (see text footnote 1) and Velvet (Zerbino et al., 2009). We also discarded five genomes due to low quality, defined by >300 contigs (*n* = 4) or <90% genome completeness (*n* = 1) for individual genomes. In addition, strain DSM 44393^T^ (GenBank accession no. CBMO000000000), ATCC 700731^T^ (GenBank accession no. JAAXPJ000000000) and JCM 14743^T^ (GenBank accession no. SRX26302896) are the same strain deposited in three type culture collections. We therefore discarded ATCC 700731^T^ and JCM 14743^T^ for further analysis.

As such, after quality control and deduplication, only 16 genomes remained including type strains *M. septicum* DSM 44393^T^ and *M. nivoides* DL90^T^. We determined the species for the 16 genomes using ANI analyses compared to *M. septicum* DSM 44393^T^ and *M. nivoides* DL90^T^ (Table 4). We found that five genomes originally labeled with *M. septicum* (ANI, 95.1 and 95.0%, with DSM 44393^T^) were actually of *M. nivoides* DL90^T^ (ANI, 99.2 and 99.2% with DL90^T^). After precise species identification, we identified that seven genomes were likely of *M. septicum*, while nine belonged to *M. nivoides* (Table 4).

**Table 4:** Average nucleotide identity (ANI) between strains labeled as *M. septicum* or *M. nivoides* and closest type strains of *Mycobacterium* species in GenBank and this study.

Strain	Accession no.	NCBI species label	Precise species	*M. septicum* DSM 44393^T^ ANI, %	*M. nivoides* DL90^T^ ANI, %	From human	Publication	Country
DSM 44393^T^^a^	CBMO000000000	*M. septicum*	*M. septicum*	100	95.2	Y	Y	France
PDNC012	CP070349	*M. septicum*	*M. septicum*	96.5	95.8	N	/	USA
WCHEs120238	JAPUCA000000000	*M. septicum*	*M. septicum*	97.0	95.4	Y	N	China
f4cb147f^b^	SRX3191976	*M. septicum*	*M. septicum*	96.3	95.0	Y	N	UK
e0f3d950^b^	SRX3192349	*M. septicum*	*M. septicum*	95.4	94.7	Y	N	UK
PSBB070	JAFIDD000000000	***M. septicum***	***M. nivoides***	95.1	99.2	N	/	USA
0881bad5^b^	SRX3192049	***M. septicum***	***M. nivoides***	95.0	99.2	Y	N	UK
A1	JARJDK000000000	*M. septicum*	*M. septicum*	95.6	94.7	N	/	Argentina
NTM040	SRX27525679	*M. septicum*	*M. septicum*	95.9	94.7	N	/	USA
CRBC_G1756	SRX26731900	***M. septicum***	***M. nivoides***	95.0	99.1	N	/	China
CRBC_G0213	SRX26721414	***M. septicum***	***M. nivoides***	95.1	99.3	N	/	China
CRBC_G1279	SRX26720797	***M. septicum***	***M. nivoides***	95.0	99.2	N	/	China
DL90^T^	CP034072	*M. nivoides*	*M. nivoides*	95.1	100	N	/	USA
88MF	SRX2140951	*M. nivoides*	*M. nivoides*	95.1	99.5	N	/	USA
455MF	SRX2140985	*M. nivoides*	*M. nivoides*	95.2	99.5	N	/	USA
120,309	JBKBDD000000000	*M. nivoides*	*M. nivoides*	94.9	99.0	Y	This study	China
120,310	JBKBDE000000000	*M. septicum*	*M. septicum*	95.7	94.6	Y	This study	China
NPDC050191	JBMVDF000000000	*M. nivoides*	*M. nivoides*	95.1	99.2	N	/	USA

a *M. septicum* DSM 44393^T^ (accession no. CBMO000000000), ATCC 700731^T^ (accession no. JAAXPJ000000000) and JCM 14743^T^ (GenBank accession no. SRX26302896) are the same strain deposited in three different type culture collections.

b f4cb147f, e0f3d950, and 0881bad5 are abbreviations of f4cb147f-c750-40fb-9178-f8005cad2b16, e0f3d950-0deb-4ace-9c9d-f0f34e57e492, and 0881bad5-9c12-4b47-b610-2e628e95d0dc, respectively.

*M. nivoides* strains that are mislabeled are shown in bold.

Y, yes; N, no.

**Figure 4:**
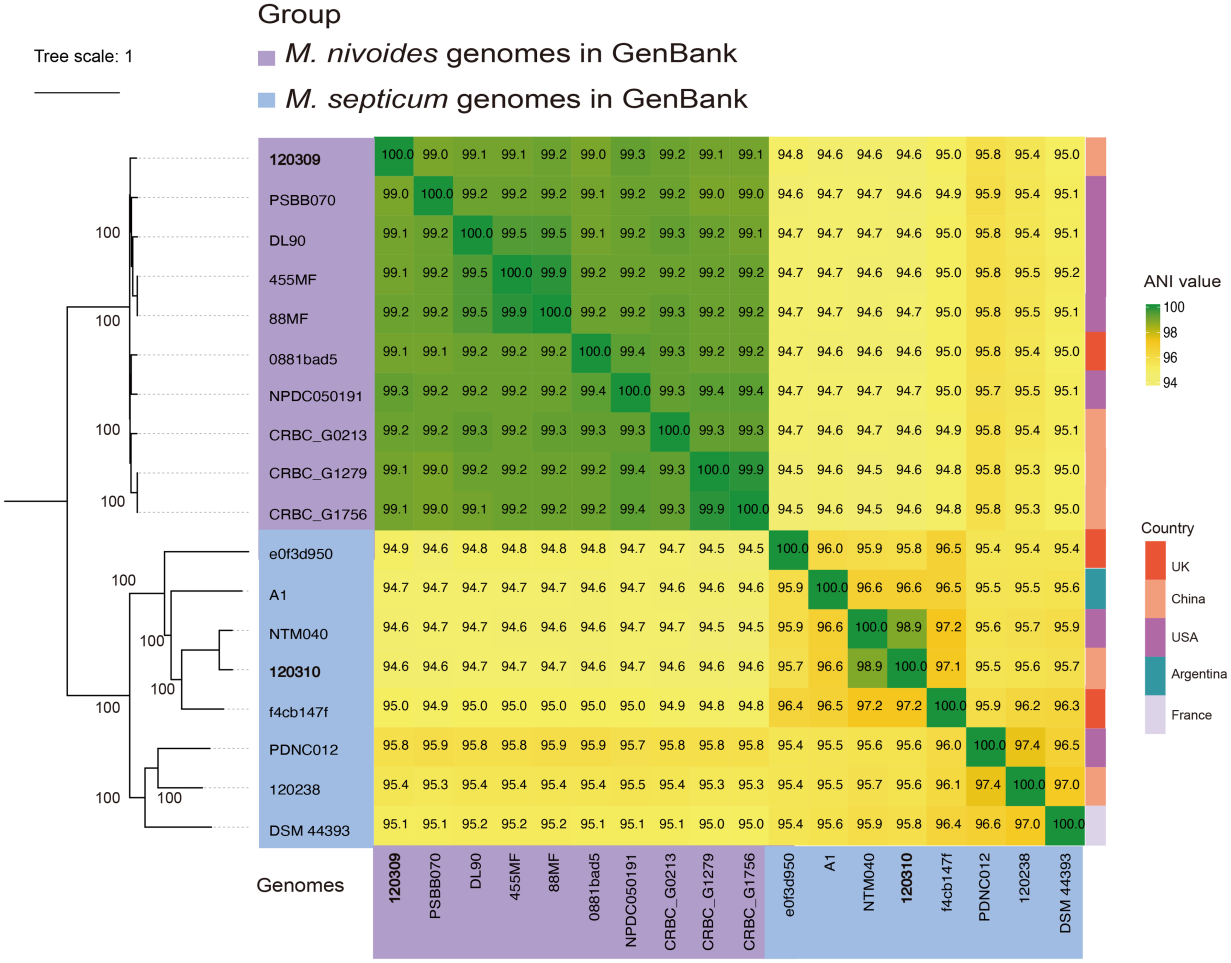
Clustering of *M. septicum* and *M. nivoides* strains in GenBank and from this study. The clustering was based on average nucleotide identity (ANI) and core SNP sites. Heat map showing pairwise ANI values for all *M. septicum* and *M. nivoides* strains. Maximum-likelihood tree of all *M. septicum* and *M. nivoides* strains was constructed based on core SNVs under GTR model with site rate variation and a 1,000-bootstrap test. The tree was inferred using strain *M. septicum* DSM 44393^T^ as the reference. The tree was midpoint rooted with bootstrap support over 50% shown in gradients. SNVs were identified via Snippy v4.6.0, aligned to the reference genomes, and filtered for recombination using Gubbins v2.4.1 under the GTR-GAMMA model. Recombination-free phylogenies were inferred with RAxML v8.2.12 using 1,000 bootstrap iterations. Trees were visualized and annotated with iTOL v6.9.

We further calculated pairwise ANI values with hierarchical clustering among the seven *M. septicum* and nine *M. nivoides* genomes together *M. nivoides* 120,309 and *M. septicum* 120,310 from this study and also constructed a SNV-based phylogenomic tree to infer their evolutionary relationships (Figure 4). The phylogenomic tree distinctly separated *M. nivoides* strains from *M. septicum*, forming two well-defined, independent branches. All seven *M. nivoides* strains clustered together with pairwise ANI values ≥ 99.0%, indicating a high genetic similarity within the species, although they were found in three countries (China, UK, and USA). In contrast, *M. septicum* strains exhibited a greater diversity with forming two distinct clades. Clade I comprises strains DSM 44393^T^ (recovered from a catheter tip), 120,238 (from sputum), and PDNC012 (from plastic debris in a land/lake environment), which shared 96.5–97.4% pairwise ANI values among themselves and 95.4–96.4% ANI values with strains in Clade II. Clade II comprised strains 120,310 (from sputum), e0f3d950-0deb-4ace-9c9d-f0f34e57e492 (from an unspecified clinical sample), f4cb147f-c750-40fb-9178-f8005cad2b16 (from an unspecified clinical sample), A1 (from soil), and NTM040 (from soil). These five strains exhibited pairwise ANI values of 95.7–97.2% among themselves and 95.4–96.3% ANI values with the aforementioned three strains in Clade I. These strains were found in 5 countries (Argentina, China, France, UK, and USA) of four continents. Our phylogenomic analysis suggests evolutionary trajectories of the *M. septicum-nivoides* complex, which comprises multiple phylogenomic groups. *M. nivoides* may diverge from the complex more recently with forming a distinct group.

We screened the sample type of the 16 genomes and found five (four *M. septicum* and one *M. nivoides*) that were recovered from clinical samples. Next, we attempted to link the five genomes to literature using two search approaches, namely direct inspection of the genome information page in National Center for Biotechnology Information (NCBI) and search of their accession numbers of Assembly, Biosample, Bioproject, and strain numbers in PubMed and Web of Science. We were only able to link only one genome to publication, which was *M. septicum* DSM 44393^T^ in a case of bloodstream infection (Schinsky et al., 2000).

## Discussion

Our study uncovers the coinfection of *M. septicum* and *M. nivoides*, two closely related species sharing an ANI > 95%, in a patient with persistent pulmonary disease. The patient’s case fulfilled the diagnostic criteria for NTM pulmonary diseases (Daley et al., 2020). Initially, MALDI-TOF MS identified *M. septicum* as the causative agent; however, WGS and ANI analyses revealed a co-infection with both *M. septicum* and *M. nivoides*. We then performed a literature review to understand the clinical relevance of the two species. We also curated the genome sequences of the two species in NCBI and uncovered misclassification of two *M. nivoides* genomes as *M. septicum*. We linked the genome sequences back to publication in attempt to provide complementary data for evaluate the clinical relevance.

*M. nivoides* has not been reported in human infection before. In our case with persistent pulmonary disease, *M. nivoides* was actually identified in two sputum samples when we sequenced 20 colonies for each sample. This fulfilled the diagnostic criteria for NTM pulmonary diseases (Daley et al., 2020) and indicates that *M. nivoides* should be regarded as a pathogen in this case. Nevertheless, considering the co-existence of *M. septicum*, whether *M. nivoides* plays a primary role or a minor one in the disease remains unclear. We note that while the patient met the clinical/radiologic/microbiological criteria for NTM pulmonary disease, the retrospective nature of the study precluded detailed longitudinal immune profiling. The consistent recovery of both species from multiple independently picked colonies across two time points supports their role as causative agents rather than contaminants or transient colonizers, but their individual pathogenic contributions cannot be ascertained. Our thorough literature review found that only two confirmed pneumonia cases attributed to *M. septicum* and almost all studies reporting *M. septicum* infection relied on MALDI-TOF MS or housekeeping gene sequencing, none of which can reliably achieve precise species identification (Adékambi and Drancourt, 2004; Mediavilla-Gradolph et al., 2015). The scarcity of reported pulmonary cases likely reflects historical diagnostic limitations rather than true rarity. Consequently, pulmonary disease caused by *M. septicum* and possibly M. nivoides is likely underreported and that the true clinical burden cannot be reliably inferred from legacy literature. Notably, although *M. septicum* is a known human pathogen with being reported in bloodstream infection, eye infection, peritonitis, and pneumonia, all but one such cases did not achieve precise species identification and misidentification cannot be excluded. As abovementioned, *M. septicum* and *M. nivoides* are closely related and are difficult, if not impossible, to be differentiated by methods used in clinical microbiology laboratories. As such, we propose that *M. septicum* should be reported as the *M. septicum*-*nivoides* complex when describing clinical cases except WGS-based precise species identification has been conducted. We suggest that for isolates identified as *M. septicum* by MALDI-TOF MS or 16S rRNA in a clinical context, laboratories should be aware of the potential for misidentification within this complex. In cases where precise identification is clinically relevant (e.g., persistent infection, epidemiological investigation), WGS-based ANI analysis should be considered to distinguish between *M. septicum* and *M. nivoides*.

The high “contamination” rate detected in strain 120,309 initially suggested sample contamination but, upon further analysis, seems to reflect genuine genomic heterogeneity. This supports the hypothesis of a simultaneous infection by multiple closely related mycobacterial strains, rather than a single clonal lineage diversifying post-infection. Therefore, tools such as CheckM may be useful to screen genomic heterogeneity, providing critical insights into such intricate infections (Parks et al., 2015).

The co-infection of *M. septicum* and *M. nivoides* with diverse clonal backgrounds in the same patient is intriguing but represents a rare and complex diagnostic challenge in NTM pulmonary diseases. Specifically, coinfection involving multiple species of NTM is rare (Hannah et al., 2020; Hill and Wooten, 2022). A review of published case reports (Hill and Wooten, 2022) has identified only a single documented case of coinfection with two closely related NTM species in an AIDS patient, where *M. avium* and *M. intracellulare*, both of which belong to the *M. avium* complex, were diagnosed using the Gen-Probe assay (Conville et al., 1989). To date, there are no studies that have explored the mechanisms underlying competitive or cooperative coexistence between closely related NTM species. Nevertheless, the coexistence of these closely related species within the same host likely reflects their ability to exploit overlapping ecological niches or shared virulence factors, suggesting potential interspecies interactions (Chevillon et al., 2024; Timme et al., 2024). These interactions could significantly influence disease pathogenicity, modulate host immune responses, and complicate clinical outcomes (Okon et al., 2023). Coinfection with two closely related NTM species and within-host genomic diversification may contribute to treatment failure or relapse by creating a broader pool of adaptive traits (e.g., divergent antimicrobial susceptibility). However, such a coexistence may reflect competitive interaction, which can benefit the affected hosts. For instance, *Staphylococcus lugdunensis* produces lugdunin, an antibiotic peptide that inhibits the colonization of *S. aureus* in the human nose (Zipperer et al., 2016). Similarly, non-pathogenic *Burkholderia thailandensis* can suppress the pathogenic *Burkholderia multivorans* through contact-dependent growth inhibition mechanisms (Myers-Morales et al., 2019). Further studies on the interspecies interactions between *M. septicum* and *M. nivoides* are warranted and may need to address both competitive and synergistic interactions, which may shed light on the ecological and pathogenic dynamics of closely related NTM species and improve understanding of their clinical significance.

This study revealed in-host microevolution in *M. septicum* and *M. nivoides*. The two species use different genetic paths toward a common adaptive strategy (enhanced virulence). *M. septicum* colonies from the second sample developed mutations of an *α*/*β* hydrolase, which have been linked to virulence (Johnson, 2017). Some *M. nivoides* colonies acquired a frameshift in *papA2,* encoding a trehalose-2-sulfate acyltransferase essential for sulfolipid-1 biosynthesis, a key virulence factor (Kumar et al., 2007). The observed virulence increase in the *G. mellonella* model provides preliminary phenotypic correlation for the identified mutations and suggests that delayed treatment may allow for adaptive changes that increase pathogenicity, highlighting the importance of prompt and precise diagnosis to initiate targeted therapy before such adaptations occur. However, definitive establishment of causality would require genetic complementation experiments in the future. Therefore, we describe these mutations as being associated with enhanced virulence in this model system.

This study has several limitations. First, the findings are based on a single patient, limiting generalizability; larger cohorts are needed to confirm the clinical significance of *M. septicum-nivoides* co-infection. Second, while *G. mellonella* provides a valuable surrogate for *in vivo* virulence assessment, its immune system differs from mammals, and virulence outcomes may not fully recapitulate human pulmonary disease. Findings should be validated in mammalian models (e.g., mouse aerosol infection) for clinical translation. Third, conventional diagnostic methods (MALDI-TOF MS, 16S rRNA sequencing) were not formally tested for distinguishing the two species, which could inform clinical workflow development. Future studies should address these gaps and explore the broader prevalence of α/β hydrolase and *papA2* mutations in NTM clinical isolates. While we propose using the ‘*M. septicum-nivoides* complex’ for laboratory reporting when WGS is unavailable (given the inability of MALDI-TOF MS or housekeeping gene sequencing to distinguish the two species), species-level resolution remains critical for clinical management*-*particularly in cases of progressive or refractory disease, epidemiological tracking, or when genomic data are accessible.

In conclusion, this study provides critical insights into the co-infection of *M. septicum* and *M. nivoides* in a patient with NTM pulmonary disease, highlighting both clinical and genomic complexities. Given the single-patient design, our conclusions are hypothesis-generating and require validation in larger cohorts. However, the findings underscore the diagnostic challenges posed by closely related species and the limitations of traditional identification methods, which can lead to misclassification and underestimation of pathogenicity. More importantly, integrated genomic and clinical analyses revealed distinct clonal lineages and genomic heterogeneity of both species, highlighting putative evolutionary strategies for long-term human adaptation.

## Data Availability

The datasets presented in this study can be found in online repositories. The names of the repository/repositories and accession number(s) can be found here: NCBI (https://www.ncbi.nlm.nih.gov/bioproject) accession number PRJNA1200829.

## Footnotes

1
https://github.com/tseemann/shovill

2
https://github.com/nanoporetech/dorado

3
https://github.com/nanoporetech/medaka/releases

4
https://github.com/gbouras13/pypolca

5
https://github.com/tseemann/snippy

6
https://www.uniprot.org

7
http://eggnog-mapper.embl.de

8
https://www.genome.jp/kegg

9
https://www.ncbi.nlm.nih.gov/genome/annotation_prok
