# Supplementary material for: Clinical significance and simultaneous microevolution of two closely-related nontuberculous mycobacteria species
Source: Front Microbiol. 2026 Apr 7;17:1791000. doi: 10.3389/fmicb.2026.1791000 (PMC13095810; doi:10.3389/fmicb.2026.1791000)
Supplement: Supplementary file 3 [file Table_1.DOCX]

**Figure S1.** Chest CT on admission revealed mild bilateral pulmonary inflammation, scattered small nodules (0.2–0.5 cm), and pleural thickening.

**
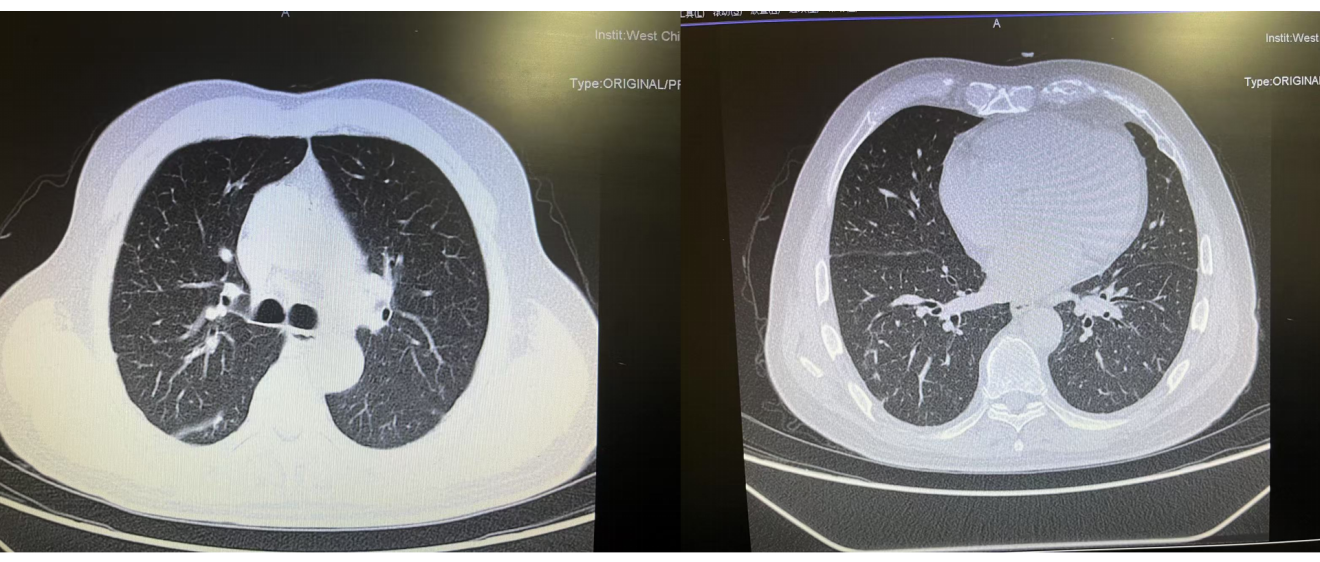
**

**Fig S2. Presence of nonsynonymous variations in *M. septicum* and *M. nivoides* colonies.**


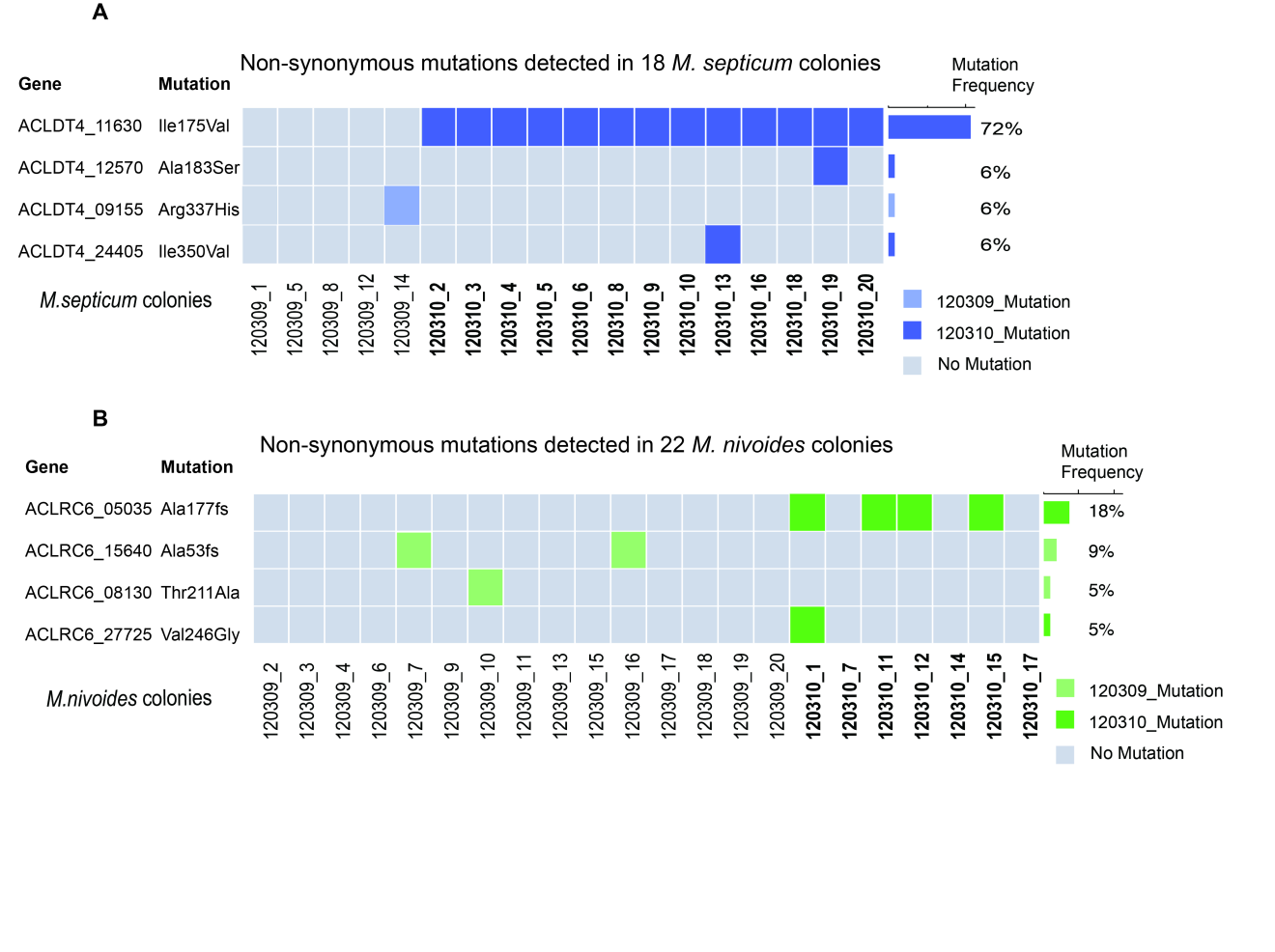


**Table S1** **Updated classification and nomenclature of *Mycobacterium* species within the “*Fortuitum-Vaccae*” clade**

| Species name^a^ | Type or reference strain | Genome accession no. | Original proposer & year |
| --- | --- | --- | --- |
| Species (n = 108)*^b^* |  |  |  |
| ***M. agri*** | CCUG 37673^T^ | PDCP00000000 | Tsukamura 1981^1^ |
| ***M. aichiense*** | NCTC 10820^T^ | UGQK00000000 | Tsukamura 1981^2^ |
| ***M. alvei*** | JCM 12272^T^ | AP022565 | Ausina et al. 1992^3^ |
| ***M. anyangense*** | JCM 30275^T^ | AP022620 | Kim et al. 2015^4^ |
| ***M. arabiense*** | JCM 18538^T^ | AP022593 | Zhang et al. 2013^5^ |
| ***M. aromaticivorans*** | JCM 16368^T^ | JALN00000000 | Hennessee et al. 2009^6^ |
| ***M. aubagnense*** | DSM 45150^T^ | POTN00000000 | Adékambi et al. 2006^7^ |
| ***M. aurantiacum*** | B3033^T^ | JAIGNG000000000 | Pan et al. 2022^8^ |
| ***M. aurum*** | NCTC 10437^T^ | CVQQ00000000 | Tsukamura 1966^9^ |
| ***M. austroafricanum*** | DSM 44191^T^ | HG964450 | Tsukamura et al. 1983^10^ |
| ***M. bacteremicum*** | DSM 45578^T^ | MVHJ00000000 | Brown-Elliott et al. 2012^11^ |
| ***M. baixiangningiae*** | LJ126^T^ | CP066218 | Cheng et al. 2021^12^ |
| ***M. boenickei*** | JCM 15653^T^ | AP022579 | Schinsky et al. 2004^13^ |
| ***M. brisbanense*** | JCM 15654^T^ | BCSX00000000 | Schinsky et al. 2004^13^ |
| ***M. brumae*** | CIP 1034565^T^ | PDCN02000000 | Luquin et al. 1993^14^ |
| ***M. canariasense*** | CCUG 47953^T^ | LQOL00000000 | Jiménez et al. 2004^15^ |
| ***M. celeriflavum*** | DSM 46765^T^ | MVHN00000000 | Shahraki et al. 2015^16^ |
| ***M. chitae*** | NCTC 10485^T^ | LR134355 | Tsukamura 1967^17^ |
| ***M. chlorophenolicum*** | JCM 7439^T^ | BCQY00000000 | Apajalahti et al. 1986^18^ |
| ***M. chubuense*** | DSM 44219^T^ | MVHO00000000 | Tsukamura 1981^2^ |
| ***M. confluentis*** | DSM 44017^T^ | LQOQ00000000 | Kirschner et al. 1992^19^ |
| ***M. cosmeticum*** | DSM 44829^T^ | CCBB000000000 | Cooksey et al. 2004^20^ |
| ***M. crocinum*** | JCM 16369^T^ | BBHD00000000 | Hennessee et al. 2009^6^ |
| ***M. diernhoferi*** | ATCC 19340^T^ | CP080332 | Tsukamura et al. 1983^10^ |
| ***M. doricum*** | DSM 44339^T^ | LQOS00000000 | Tortoli et al. 2001^21^ |
| ***M. duvalii*** | JCM 6396^T^ | AP022563 | Stanford and Gunthorpe 1971^22^ |
| ***M. elephantis*** | DSM 44368^T^ | JACKTZ000000000 | Shojaei et al. 2000^23^ |
| ***M. fallax*** | DSM 44179^T^ | LQOJ00000000 | Lévy-Frébault et al. 1983^24^ |
| ***M. farcinogenes*** | DSM 43637^T^ | CCAY000000000^k^ | Chamoiseau 1973^25^ |
| ***M. flavescens*** | DSM 43991^T^ | JACKUL000000000 | Bojalil et al. 1962^26^ |
| ***M. fluoranthenivorans*** | DSM 44556^T^ | JAANOW000000000 | Hormisch et al. 2006^27^ |
| ***M. fortuitum*** | JCM 6387^T^ | AP025518 | da Costa Cruz 1938^28^ |
| ***M. frederiksbergense*** | DSM 45364^T^ | JACKTH000000000 | Willumsen et al. 2001^29^ |
| ***M. gadium*** | JCM 12688^T^ | AP022608 | Casal and Calero 1974^30^ |
| ***M. gilvum*** | NCTC 10742^T^ | UGQM00000000 | Stanford and Gunthorpe 1971^22^ |
| *M. goodii* | ATCC 700504^T^ | CP092364 | Brown et al. 1999^31^ |
| ***M. gossypii*** | S2-37^T^ | JAFEVR010000015 | Huang et al. 2021^32^ |
| ***M. hassiacum*** | DSM 44199^T^ | KB903840 | Schröder et al. 1997^33^ |
| ***M. helvum*** | JCM 30396^T^ | AP022596 | Tran and Dahl 2016^34^ |
| ***M. hippocampi*** | DSM 45391^T^ | JACKSE000000000 | Balcázar et al. 2014^35^ |
| ***M. hodleri*** | JCM 12141^T^ | BBGO00000000 | Kleespies et al. 1996^36^ |
| ***M. holsaticum*** | JCM 12374^T^ | CP080998 | Richter et al. 2002^37^ |
| ***M. houstonense*** | ATCC 49403^T^ | FJVO00000000 | Schinsky et al. 2004^13^ |
| ***M. insubricum*** | DSM 45132^T^ | MVHS00000000 | Tortoli et al. 2009^38^ |
| ***M. iranicum*** | DSM 45541^T^ | LQPC00000000 | Shojaei et al. 2013^39^ |
| ***M. komaniense*** | GPK 1020^T^ | CVTA00000000 | Gcebe et al. 2018^40^ |
| ***M. komossense*** | DSM 44078^T^ | JACKTY000000000 | Kazda and Müller 1979^41^ |
| ***M. lacusdiani*** | JXJ CY 35^T^ | JAKCFC000000000 | Xiao et al. 2023^42^ |
| ***M. litorale*** | JCM 17423^T^ | AP022586 | Zhang et al. 2012^43^ |
| ***M. llatzerense*** | MG13^T^ | LXOV00000000 | Gomila et al. 2008^44^ |
| ***M. lutetiense*** | DSM 46713^T^ | JAGIOP000000000 | Konjek et al. 2016^45^ |
| ***M. madagascariense*** | JCM 13574^T^ | AP022610 | Kazda et al. 1992^46^ |
| ***M. mageritense*** | CIP 104973^T^ | CCBF000000000 | Domenech et al. 1997^47^ |
| ***M. malmesburyense*** | WCM 7299^T^ | CVTB00000000 | Gcebe et al. 2017^48^ |
| ***M. mengxianglii*** | Z-34^T^ | CP065373 | Cheng et al. 2021^12^ |
| ***M. monacense*** | DSM 44395^T^ | MVIA00000000 | Reischl et al. 2006^49^ |
| ***M. moriokaense*** | JCM 6375^T^ | AP022560 | Tsukamura et al. 1986^50^ |
| ***M. mucogenicum*** | CSUR P2099^f^ | CYSI00000000 | Springer et al. 1995^51^ |
| ***M. neoaurum*** | ATCC 25795^T^ | JMDW00000000 | Tsukamura 1972^52^ |
| ***M. neworleansense*** | ATCC 49404^T^ | CWKH00000000 | Schinsky et al. 2004^13^ |
| ***M. nivoides*** | DL90^T^ | CP034072 | Dahl et al. 2021^53^ |
| ***M. novocastrense*** | JCM 18114^T^ | BCTA00000000 | Shojaei et al. 1997^54^ |
| ***M. obuense*** | DSM 44075^T^ | JYNU00000000 | Tsukamura and Mizuno 1981^2^ |
| ***M. pallens*** | JCM 16370^T^ | BBHE00000000 | Hennessee et al. 2009^6^ |
| ***M. parafortuitum*** | CCUG 20999^T^ | MVID00000000 | Tsukamura 1966^55^ |
| ***M. peregrinum*** | DSM 43271^T^ | LQPP00000000 | Kusunoki and Ezaki 1992^56^ |
| ***M. phlei*** | CCUG 21000^T^ | CP014475 | Lehmann and Neumann 1899^57^ |
| ***M. phocaicum*** | DSM 45104^T^ | POTM00000000 | Adékambi et al. 2006^7^ |
| ***M. porcinum*** | DSM 44242^T^ | JACKVC000000000 | Tsukamura et al. 1983^58^ |
| ***M. poriferae*** | JCM 12603^T^ | AP022570 | Padgitt and Moshier 1987^59^ |
| ***M. psychrotolerans*** | JCM 13323^T^ | AP022574 | Trujillo et al. 2004^60^ |
| ***M. pulveris*** | JCM 6370^T^ | AP022599 | Tsukamura et al. 1983^61^ |
| ***M. pyrenivorans*** | JCM 15927^T^ | BBHB00000000 | Derz et al. 2004^62^ |
| ***M. rhodesiae*** | DSM 44223^T^ | MVIH00000000 | Tsukamura 1981^2^ |
| ***M. rufum*** | JCM 16372^T^ | BBGS00000000 | Hennessee et al. 2009^6^ |
| ***M. rutilum*** | JCM 16371^T^ | BBHF00000000 | Hennessee et al. 2009^6^ |
| ***M. sarraceniae*** | JCM 30395^T^ | AP022595 | Tran and Dahl 2016^34^ |
| ***M. sediminis*** | DSM 45643^T^ | JACKUW000000000 | Zhang et al. 2013^5^ |
| ***M. senegalense*** | ATCC 35796^T^ | CP081000 | Chamoiseau 1973^63^ |
| ***M. septicum*** | DSM 44393^T^ | CBMO000000000 | Schinsky et al. 2000^64^ |
| ***M. setense*** | DSM 45070^T^ | JTJW00000000 | Lamy et al. 2008^65^ |
| ***M. smegmatis*** | NCTC 8159^T^ | LN831039 | Lehmann and Neumann 1899^57^ |
| ***M. sphagni*** | ATCC 33027^T^ | NOZR00000000 | Kazda 1980^66^ |
| ***M. stellerae*** | CECT 8783^T^ | RARC00000000 | Nouioui et al. 2019^67^ |
| ***M. thermoresistibile*** | ATCC 19527^T^ | AGVE00000000 | Tsukamura 1966^9^ |
| ***M. tokaiense*** | NCTC10821^T^ | UGQT00000000 | Tsukamura 1981^2^ |
| ***M. tusciae*** | DSM 44338^T^ | MVIM00000000 | Tortoli et al. 1999^68^ |
| ***M. vaccae*** | ATCC 25954^T^ | JH814683 | Bönicke and Juhasz 1964^69^ |
| ***M. vinylchloridicum*** | CECT 8761^T^ | JACBJQ000000000 | Cortés-Albayay et al. 2023^70^ |
| ***M. wolinskyi*** | ATCC 700010^T^ | LQQA00000000 | Brown et al. 1999^31^ |
| ***M. xanthum*** | Y57^T^ | JAIFZS000000000 | Pan et al. 2022^8^ |
| ***M. aquaticum^d^*** | RW6^T^ | MVHF00000000 | Hashemi Shahraki et al. 2017^71^ |
| *M. barrassiae^d^* | CCUG 50398^T^ | JACKUK010000001 | Adékambi et al. 2006^72^ |
| *M. dioxanotrophicus^d^* | PH-06^T^ | CP020809 | He et al. 2017^73^ |
| *M. fortunisiensis^d^* | TNTM28^T^ | VOMB01000005 | Gharbi et al. 2021^74^ |
| *M. gallinarum^d^* | JCM 6399^f^ | AP022601 | Tsukamura et al. 1967^75^ |
| *M. grossiae^d^* | DSM 104744^T^ | CP043474 | Paniz-Mondolfi et al. 2017^76^ |
| *M. hackensackense^d^* | DSM 44833^T^ | JACKUC010000001 | Hong et al. 2003^77^ |
| ***M. kyogaense^d^*** | NCTC 11659^T^ | QJUA00000000 | Nouioui et al. 2018^78^ |
| ***M. lehmannii^d^*** | CECT 8763^T^ | NKCN00000000 | Nouioui et al. 2017^79^ |
| *M. manitobense^d^* | DSM 44615^T^ | JACKSJ010000001 | Turenne et al. 2003^80^ |
| *M. massilipolynesiensis^d^* | M26^T^ | LN929900 | Phelippeau et al. 2017^81^ |
| ***M. neglectum^d^*** | CECT 8778^T^ | NVQE00000000 | Nouioui et al. 2019^82^ |
| ***M. neumannii^d^*** | CECT 8766^T^ | NKCO00000000 | Nouioui et al. 2017^79^ |
| ***M. palauense^d^*** | CECT 8779^T^ | NVQF00000000 | Nouioui et al. 2019^82^ |
| *M. salfingeri^d^* | 20-157661^T^ | CP081006 | Musser et al. 2022^83^ |
| ***M. syngnathidarum^d^*** | 27335^T^ | MLCL00000000 | Fogelson et al. 2018^84^ |
| *M. yunnanensis^d^* | DSM 44838^T^ | JACKVK010000001 | Li et al. |
| Species rejected (n=4) | | | |
| *M. vanbaalenii^g^* | PYR-1^T^ | *M. austroafricanum* |  |
| *M. conceptionense^h^* | CCUG 50187^T^ | *M. senegalense* |  |
| *M. murale^i^* | JCM 13392^T^ | *M. tokaiense* |  |
| *M. acapulcensis^c^* | CSURP1424^e^ | *M. lehmannii* |  |
| Species listed in LPSN but moved out of FVC (n=1) | | | |
| *M. vulneris^j^* | DSM 45247^T^ | [NCXM01000000](https://www.ncbi.nlm.nih.gov/nuccore/NCXM01000000) |  |

^a^Gupta et al. proposed in 2018^85^ to transfer species of the "*Fortuitum-Vaccae*" clade from the genus *Mycobacterium* to a new genus named *Mycolicibacterium.* The new species names with *Mycolicibacterium* have been included in a validation list by IJSEM^86^. However, Tortoli et al^87^ and [Meehan](https://www.microbiologyresearch.org/search?value1=Conor+J.+Meehan&option1=author&noRedirect=true) et al^88^ proposed to keep using the original names with *Mycobacterium*, given that the new nomenclature has the potential to cause confusion and provides no benefits to the field of clinical mycobacteriology. The original names remain validly published, and anyone is free to use them^89^. In our study, we use the original names.

^b^The species in bold are validly published and those underlined are effectively published, while those neither in bold nor underlined have not been validly or effectively published.

^c^*M. acapulcensis* was renamed *M. acapulense* by Gupta et al^85^.

^d^*M. aquaticum, M. barrassiae, M. dioxanotrophicus, M. fortunisiensis, M. gallinarum, M. grossiae, M. hackensackense, M. kyogaense, M. lehmannii, M. manitobense, M. massilipolynesiensis, M. neglectum, M. neumannii, M. palauense, M. salfingeri, M. syngnathidarum,* and *M. yunnanensis* have not been included in the study by Gupta et al^85^.

^e^As the genome of the type strain of *M. acapulcensis* is not available in GenBank, we used genome of strain CSURP1424, which has been published previously^90^ as the reference strain.

^f^As the genome of the type strain of *M. mucogenicum*, *M. alkanivorans* and *M. gallinarum* are not available in GenBank, we used genome of strain CSUR P2099, ANDR5 and JCM 6399, which were deposited in GenBank at the earliest date among all genomes labeled *M. mucogenicum*, *M. alkanivorans* and *M. gallinarum* as the reference strain, respectively.

*^g^M. vanbaalenii* is proposed to be a later heterotypic synonym of *M. austroafricanum*^91^.

^h^*M. conceptionense* is proposed to be a later heterotypic synonym of *M. senegalense*^91^.

^I^*M. murale* is proposed to be a later heterotypic synonym of *M. tokaiense* based on genome analysis in this study.

^j^[Tortoli](https://pubmed.ncbi.nlm.nih.gov/?term=Tortoli%20E%5bAuthor%5d)^87^ and Gupta et al^92^ have reported that the type strain of *Mycolicibacterium vulneris*, DSM 45247^T^ (GenBank accession no. [NCXM01000000](https://www.ncbi.nlm.nih.gov/nuccore/NCXM01000000)) is clusterd within the slow-growing group of mycobacteria, and proposed that *Mycolicibacterium vulneris* (Gupta et al., [2018](#B2)) should be reinstated to its previous basonym *Mycobacterium vulneris* (van Ingen et al., [2009](#B4)) as a slow grower. Therefore, *M. vulneris* labled as “*Mycolicibacterium*” in LPSN is not included in this table.

^k^It has been pointed out previously^93^ that the genome of *Mycobacterium farcinogenes* type strain DSM 43637^T^ (accession no. CCAY000000000) is actually obtained from a strain of *Mycobacterium senegalense.*

**Table S2. Survival of *Galleria mellonella*larva infected with the paren**tal ***M. septicum* strain 120309_1 and α/β hydrolase mu**tant 120310_9

| Strain | Group | Bacterial load, CFU/mL,  0.01 mL | Replicate | No. of survival (Time, post-infection) | | | | | | | | | |
| --- | --- | --- | --- | --- | --- | --- | --- | --- | --- | --- | --- | --- | --- |
|  |  |  |  | 0 h | 0.5 h | 24 h | 48 h | 72 h | 96 h | 120 h | 144 h | 216 h | 264 h |
|  | PBS | / | 1^st^ | 16 | 16 | 15 | 15 | 15 | 15 | 15 | 15 | 15 | 15 |
|  |  |  | 2^nd^ | 16 | 16 | 15 | 15 | 15 | 15 | 15 | 15 | 15 | 15 |
|  |  |  | 3^rd^ | 16 | 16 | 16 | 16 | 15 | 15 | 15 | 14 | 14 | 14 |
| parental strain 120309_1 | Load 1 | 5×10^10^ | 1^st^ | 16 | 0 | 0 | 0 | 0 | 0 | 0 | 0 | 0 | 0 |
|  | Load 2 | 2.5×10^10^ | 1^st^ | 16 | 0 | 0 | 0 | 0 | 0 | 0 | 0 | 0 | 0 |
|  | Load 3 | 5×10^9^ | **1^st^** | **16** | **16** | **12** | **11** | **10** | **8** | **6** | **3** | **3** | **3** |
|  |  |  | **2^nd^** | **16** | **16** | **12** | **12** | **11** | **10** | **9** | **8** | **7** | **6** |
|  |  |  | **3^rd^** | **16** | **16** | **13** | **12** | **9** | **7** | **6** | **5** | **4** | **3** |
|  |  |  | **overall** | **48** | **48** | **37** | **35** | **30** | **25** | **21** | **16** | **14** | **12** |
|  | Load 4 | 1.5×10^9^ | 1^st^ | 16 | 16 | 16 | 15 | 13 | 12 | 12 | 12 | 12 | 11 |
|  |  |  | 2^nd^ | 16 | 16 | 15 | 14 | 14 | 13 | 11 | 11 | 11 | 11 |
|  |  |  | 3^rd^ | 16 | 16 | 15 | 14 | 14 | 14 | 14 | 14 | 14 | 13 |
|  |  |  | overall | 48 | 48 | 46 | 43 | 41 | 39 | 37 | 37 | 37 | 35 |
|  | Load 5 | 7.5×10^8^ | 1^st^ | 16 | 16 | 16 | 15 | 15 | 15 | 15 | 15 | 14 | 13 |
|  |  |  | 2^nd^ | 16 | 16 | 16 | 16 | 16 | 15 | 15 | 15 | 15 | 14 |
|  |  |  | 3^rd^ | 16 | 16 | 16 | 16 | 16 | 15 | 15 | 15 | 14 | 14 |
|  |  |  | overall | 48 | 48 | 48 | 47 | 47 | 45 | 45 | 45 | 43 | 41 |
| *M. septicum* α/β hydrolase mutant 120310_9 | Load 1 | 5×10^10^ |  | 16 | 0 | 0 | 0 | 0 | 0 | 0 | 0 | 0 | 0 |
|  | Load 2 | 2.5×10^10^ |  | 16 | 0 | 0 | 0 | 0 | 0 | 0 | 0 | 0 | 0 |
|  | Load 3 | 5×10^9^ | **1^st^** | **16** | **16** | **10** | **8** | **6** | **5** | **2** | **2** | **1** | **1** |
|  |  |  | **2^nd^** | **16** | **16** | **11** | **8** | **7** | **6** | **4** | **4** | **4** | **4** |
|  |  |  | **3^rd^** | **16** | **16** | **9** | **6** | **5** | **5** | **4** | **4** | **3** | **3** |
|  |  |  | **overall** | **48** | **48** | **30** | **22** | **18** | **16** | **10** | **10** | **8** | **8** |
|  | Load 4 | 1.5×10^9^ | 1^st^ | 16 | 16 | 16 | 14 | 14 | 12 | 10 | 9 | 9 | 9 |
|  |  |  | 2^nd^ | 16 | 16 | 15 | 13 | 13 | 13 | 11 | 10 | 10 | 10 |
|  |  |  | 3^rd^ | 16 | 16 | 14 | 14 | 13 | 13 | 13 | 12 | 12 | 11 |
|  |  |  | overall | 48 | 48 | 45 | 41 | 40 | 38 | 34 | 31 | 31 | 30 |
|  | Load 5 | 7.5×10^8^ | 1^st^ | 16 | 16 | 16 | 15 | 15 | 15 | 13 | 13 | 12 | 12 |
|  |  |  | 2^nd^ | 16 | 16 | 15 | 15 | 14 | 14 | 14 | 14 | 13 | 13 |
|  |  |  | 3^rd^ | 16 | 16 | 16 | 16 | 15 | 15 | 14 | 14 | 14 | 14 |
|  |  |  | overall | 48 | 48 | 47 | 46 | 44 | 44 | 41 | 41 | 39 | 39 |

**Table S3. Survival of *Galleria mellonella*larva infected with the parental *M. nivoides* strain 120309_2 and *papA2* mutant 120310_11**

| Strain | Group | Bacterial load, CFU/mL,  0.01 mL | Replicate | No. of survival (Time, post-infection) | | | | | | | | | |
| --- | --- | --- | --- | --- | --- | --- | --- | --- | --- | --- | --- | --- | --- |
|  |  |  |  | 0 h | 0.5 h | 24 h | 48 h | 72 h | 96 h | 120 h | 144 h | 216 h | 264 h |
|  | PBS | / | 1^st^ | 16 | 16 | 16 | 15 | 15 | 15 | 15 | 15 | 14 | 14 |
|  |  |  | 2^nd^ | 16 | 16 | 15 | 15 | 15 | 15 | 15 | 15 | 15 | 15 |
|  |  |  | 3^rd^ | 16 | 16 | 16 | 16 | 16 | 16 | 15 | 15 | 15 | 15 |
| parental strain 120309_2 | Load 1 | 5×10^10^ | 1^st^ | 16 | 0 | 0 | 0 | 0 | 0 | 0 | 0 | 0 | 0 |
|  | Load 2 | 2.5×10^10^ | 1^st^ | 16 | 0 | 0 | 0 | 0 | 0 | 0 | 0 | 0 | 0 |
|  | Load 3 | 5×10^9^ | **1^st^** | **16** | **16** | **14** | **14** | **8** | **5** | **4** | **4** | **3** | **3** |
|  |  |  | **2^nd^** | **16** | **16** | **16** | **13** | **5** | **2** | **2** | **2** | **2** | **2** |
|  |  |  | **3^rd^** | **16** | **16** | **16** | **10** | **4** | **3** | **3** | **3** | **3** | **3** |
|  |  |  | **overall** | **48** | **48** | **46** | **37** | **17** | **10** | **9** | **9** | **8** | **8** |
|  | Load 4 | 1.5×10^9^ | 1^st^ | 16 | 16 | 15 | 15 | 14 | 14 | 13 | 13 | 10 | 6 |
|  |  |  | 2^nd^ | 16 | 16 | 16 | 16 | 16 | 16 | 16 | 16 | 15 | 12 |
|  |  |  | 3^rd^ | 16 | 16 | 16 | 16 | 16 | 16 | 16 | 16 | 16 | 14 |
|  |  |  | overall | 48 | 48 | 47 | 47 | 46 | 46 | 45 | 45 | 41 | 32 |
|  | Load 5 | 7.5×10^8^ | 1^st^ | 16 | 16 | 16 | 16 | 15 | 15 | 15 | 15 | 14 | 10 |
|  |  |  | 2^nd^ | 16 | 16 | 16 | 16 | 16 | 16 | 16 | 16 | 15 | 12 |
|  |  |  | 3^rd^ | 16 | 16 | 16 | 16 | 16 | 16 | 16 | 16 | 15 | 15 |
|  |  |  | overall | 48 | 48 | 48 | 48 | 47 | 47 | 47 | 47 | 44 | 37 |
| *M. nivoides papA2* mutant 120310_11 | Load 1 | 5×10^10^ |  | 16 | 0 | 0 | 0 | 0 | 0 | 0 | 0 | 0 | 0 |
|  | Load 2 | 2.5×10^10^ |  | 16 | 0 | 0 | 0 | 0 | 0 | 0 | 0 | 0 | 0 |
|  | Load 3 | 5×10^9^ | **1^st^** | **16** | **16** | **12** | **7** | **3** | **3** | **1** | **1** | **1** | **1** |
|  |  |  | **2^nd^** | **16** | **16** | **13** | **5** | **3** | **2** | **1** | **1** | **1** | **1** |
|  |  |  | **3^rd^** | **16** | **16** | **13** | **5** | **4** | **4** | **3** | **3** | **2** | **2** |
|  |  |  | **overall** | **48** | **48** | **38** | **17** | **10** | **9** | **5** | **5** | **4** | **4** |
|  | Load 4 | 1.5×10^9^ | 1^st^ | 16 | 16 | 16 | 16 | 16 | 16 | 16 | 16 | 13 | 11 |
|  |  |  | 2^nd^ | 16 | 16 | 16 | 16 | 16 | 16 | 15 | 15 | 12 | 10 |
|  |  |  | 3^rd^ | 16 | 16 | 15 | 15 | 14 | 14 | 14 | 14 | 13 | 6 |
|  |  |  | overall | 48 | 48 | 47 | 47 | 46 | 46 | 45 | 45 | 38 | 27 |
|  | Load 5 | 7.5×10^8^ | 1^st^ | 16 | 16 | 16 | 16 | 16 | 16 | 16 | 16 | 13 | 9 |
|  |  |  | 2^nd^ | 16 | 16 | 16 | 16 | 16 | 16 | 16 | 16 | 15 | 9 |
|  |  |  | 3^rd^ | 16 | 16 | 16 | 16 | 15 | 15 | 15 | 15 | 15 | 10 |
|  |  |  | overall | 48 | 48 | 48 | 48 | 47 | 47 | 47 | 47 | 43 | 28 |

**Table S4. Survival of *Galleria mellonella*larva infected with the parental *M. septicum* strain 120309_1 and α/β hydrolase mutant 120310_9 with significant inter-group differences occurring at 10 μL of 5 × 10^9^ CFU/mL**

| Time, post-infection | Bacterial load, CFU/mL,  0.01 mL | parental strain 120309_1 | | | | *M. septicum* α/β hydrolase mutant 120310_9 | | | | PBS | | | |
| --- | --- | --- | --- | --- | --- | --- | --- | --- | --- | --- | --- | --- | --- |
|  |  | 1^st^ | 2^nd^ | 3^rd^ | overall (%) | 1^st^ | 2^nd^ | 3^rd^ | overall (%) | 1^st^ | 2^nd^ | 3^rd^ | overall (%) |
| 0 h | 5×10^9^ | 16 | 16 | 16 | 48 (100%) | 16 | 16 | 16 | 48 (100%) | 16 | 16 | 16 | 48 (100%) |
| 24 h |  | 12 | 12 | 13 | 37 (77.08%) | 10 | 11 | 9 | 30 (62.50%) | 15 | 15 | 16 | 46 (95.83%) |
| 48 h |  | 11 | 12 | 12 | **35 (72.92%)** | 8 | 8 | 6 | **22 (45.83%)** | 15 | 15 | 16 | 46 (95.83%) |
| **72 h** |  | 10 | 11 | 9 | **30 (62.50%)** | 6 | 7 | 5 | **18 (37.50%)** | 15 | 15 | 15 | 45 (93.75%) |
| 96 h |  | 8 | 10 | 7 | 25 (52.08%) | 5 | 6 | 5 | 16 (33.33%) | 15 | 15 | 15 | 45 (93.75%) |
| 120 h |  | 6 | 9 | 6 | 21 (43.75%) | 2 | 4 | 4 | 10 (20.83%) | 15 | 15 | 15 | 45 (93.75%) |
| 144 h |  | 3 | 8 | 5 | 16 (33.33%) | 2 | 4 | 4 | 10 (20.83%) | 15 | 15 | 14 | 44 (91.67%) |
| 216 h |  | 3 | 7 | 4 | 14 (29.16%) | 1 | 4 | 3 | 8 (16.70%) | 15 | 15 | 14 | 44 (91.67%) |
| 264 h |  | 3 | 6 | 3 | 12 (25.00%) | 1 | 4 | 3 | 8 (16.70%) | 15 | 15 | 14 | 44 (91.67%) |

**Table S5. Survival of *Galleria mellonella*larva infected with the parental *M. nivoides* strain 120309_2 and *papA2* mutant 120310_11 with significant inter-group differences occurring at 10 μL of 5 × 10^9^ CFU/mL**

| Time, post-infection | Bacterial load, CFU/mL,  0.01 mL | parental strain 120309_2 | | | | *M. nivoides* papA2 mutant 120310_11 | | | | PBS | | | |
| --- | --- | --- | --- | --- | --- | --- | --- | --- | --- | --- | --- | --- | --- |
|  |  | 1^st^ | 2^nd^ | 3^rd^ | overall (%) | 1^st^ | 2^nd^ | 3^rd^ | overall (%) | 1^st^ | 2^nd^ | 3^rd^ | overall (%) |
| 0 h | 5×10^9^ | 16 | 16 | 16 | 48 (100%) | 16 | 16 | 16 | 48 (100%) | 16 | 16 | 16 | 48 (100%) |
| 24 h |  | 14 | 16 | 16 | 46 (95.83%) | 12 | 13 | 13 | 38 (79.16%) | 16 | 15 | 16 | 47 (97.92%) |
| **48 h** |  | 14 | 13 | 10 | **37 (77.08%)** | 7 | 5 | 5 | **17 (35.42%)** | 15 | 15 | 16 | **46 (95.83%)** |
| 72 h |  | 8 | 5 | 4 | 17 (35.42%) | 3 | 3 | 4 | 10 (20.83%) | 15 | 15 | 16 | 46 (95.83%) |
| 96 h |  | 5 | 2 | 3 | 10 (20.83%) | 3 | 2 | 4 | 9 (18.75%) | 15 | 15 | 16 | 46 (95.83%) |
| 120 h |  | 4 | 2 | 3 | 9 (18.75%) | 1 | 1 | 3 | 5 (10.42%) | 15 | 15 | 15 | 45 (93.75%) |
| 144 h |  | 4 | 2 | 3 | 9 (18.75%) | 1 | 1 | 3 | 5 (10.42%) | 15 | 15 | 15 | 45 (93.75%) |
| 216 h |  | 3 | 2 | 3 | 8 (16.67%) | 1 | 1 | 2 | 4 (8.33%) | 14 | 15 | 15 | 44 (91.67%) |
| 264 h |  | 3 | 2 | 3 | 8 (16.67%) | 1 | 1 | 2 | 4 (8.33%) | 14 | 15 | 15 | 44 (91.67%) |

**Table S6. Antimicrobial resistance profiles and MIC values of isolates 120309 and 120310**

| **Antimicrobial Agent** | **MIC** | |
| --- | --- | --- |
|  | **120309** | **120310** |
| Doxycycline | 128 | 128 |
| Imipenem | >64 | >64 |
| Rifampin | 16 | 16 |
| Rifabutin | 8 | 8 |
| Sulfamethoxazole | 256 | 256 |
| Amikacin | 4 | 4 |
| Gatifloxacin | 0.06 | 0.06 |
| Moxifloxacin | 0.125 | 0.125 |
| Linezolid | 8 | 8 |
| Cefoxitin | 32 | 32 |
| Azithromycin | >32* | >32* |
| Minocycline | 32 | 128 |
| Tobramycin | 8 | 8 64 |
| Ethambutol | 5* | 10* |
| Clarithromycin | 4 | 64 |

*no CLSI-defined breakpoints for azithromycin and ethambutol.

**Table S7. Pairwise ANI analysis of *M. septicum*, *M. nivoides* and other *Mycobacterium* species in the same branch of the phylogenomic tree**

|  | *M.*  *alvei* | *M.*  *boenickei* | *M.*  *fortuitum* | *M.*  *lutetiense* | *M.*  *neworleansense* | *M.*  *nivoides* | *M.*  *peregrinum* | *M.*  *porcinum* | *M.*  *senegalense* | *M.*  *septicum* | *M.*  *setense* | *M.*  *syngnathidarum* |
| --- | --- | --- | --- | --- | --- | --- | --- | --- | --- | --- | --- | --- |
| *M. alvei* | / | 86.9 | 86.5 | 92.0 | 86.8 | 87.8 | 88.0 | 86.7 | 86.0 | 87.7 | 86.2 | 86.0 |
| *M. boenickei* | 86.8 | / | 87.0 | 86.7 | 88.6 | 89.2 | 87.6 | 93.0 | 88.3 | 89.2 | 87.5 | 88.2 |
| *M. fortuitum* | 86.3 | 87.0 | / | 86.3 | 87.0 | 86.9 | 88.1 | 87.1 | 87.3 | 87.0 | 85.8 | 86.0 |
| *M. lutetiense* | 91.9 | 86.9 | 86.4 | / | 87.0 | 87.5 | 87.9 | 86.6 | 86.0 | 87.7 | 86.2 | 86.5 |
| *M. neworleansense* | 86.7 | 88.6 | 87.0 | 86.8 | / | 88.8 | 87.2 | 88.3 | 87.5 | 89.1 | 87.5 | 87.3 |
| *M. nivoides* | 87.5 | 89.2 | 86.9 | 87.5 | 88.8 | / | 87.8 | 88.8 | 87.4 | **95.1** | 88.1 | 87.6 |
| *M. peregrinum* | 87.7 | 87.6 | 88.0 | 87.8 | 87.3 | 87.9 | / | 87.5 | 86.9 | 88.2 | 86.4 | 86.7 |
| *M. porcinum* | 86.5 | 93.0 | 87.1 | 86.5 | 88.2 | 88.9 | 87.5 | / | 88.2 | 88.9 | 87.2 | 87.8 |
| *M. senegalense* | 85.9 | 88.4 | 87.3 | 85.9 | 87.5 | 87.5 | 87.0 | 88.3 | / | 87.7 | 86.5 | 89.2 |
| *M. septicum* | 87.6 | 89.2 | 86.9 | 87.5 | 89.0 | **95.2** | 88.2 | 88.9 | 87.5 | / | 88.1 | 87.6 |
| *M. setense* | 86.1 | 87.5 | 85.8 | 86.2 | 87.5 | 88.1 | 86.4 | 87.3 | 86.4 | 88.0 | / | 86.5 |
| *M. syngnathidarum* | 85.9 | 88.2 | 86.2 | 86.5 | 87.4 | 87.6 | 86.6 | 87.9 | 89.2 | 87.6 | 86.6 | / |

Those ≥95% are in bold.

**References**

1. Tsukamura, M. Numerical analysis of rapidly growing, nonphotochromogenic mycobacteria, including *Mycobacterium agri* (Tsukamura 1972) Tsukamura sp. nov., nom. rev. *Int J Syst Bacteriol* **31**, 247-258 (1981).

2. Tsukamura, M., Mizuno, S. & Tsukamura, S. Numerical analysis of rapidly growing, scotochromogenic mycobacteria, including *Mycobacterium obuense* sp. nov., nom. rev., *Mycobacterium rhodesiae* sp. nov., nom. rev., *Mycobacterium aichiense* sp. nov., nom. rev., *Mycobacterium chubuense* sp. nov., nom. rev., and *Mycobacterium tokaiense* sp. nov., nom. rev. *Int J Syst Evol Microbiol* **31**, 263-275 (1981).

3. Ausina, V. et al. *Mycobacterium alvei* sp. nov. *Int J Syst Bacteriol* **42**, 529-535 (1992).

4. Kim, B. J. et al. *Mycobacterium anyangense* sp. nov., a rapidly growing species isolated from blood of Korean native cattle, Hanwoo (Bos taurus coreanae). *Int J Syst Evol Microbiol* **65**, 2277-2285 (2015).

5. Zhang, D. F. et al. *Mycobacterium sediminis* sp. nov. and *Mycobacterium arabiense* sp. nov., two rapidly growing members of the genus *Mycobacterium*. *Int J Syst Evol Microbiol* **63**, 4081-4086 (2013).

6. Hennessee, C. T. et al. Polycyclic aromatic hydrocarbon-degrading species isolated from Hawaiian soils: *Mycobacterium crocinum* sp. nov., *Mycobacterium pallens* sp. nov., *Mycobacterium rutilum* sp. nov., *Mycobacterium rufum* sp. nov. and *Mycobacterium aromaticivorans* sp. nov. *Int J Syst Evol Microbiol* **59**, 378-387 (2009).

7. Adekambi, T. et al. *rpoB* gene sequence-based characterization of emerging non-tuberculous mycobacteria with descriptions of *Mycobacterium bolletii* sp. nov., *Mycobacterium phocaicum* sp. nov., and *Mycobacterium aubagnense* sp. nov. *Int J Syst Evol Microbiol* **56**, 133-143 (2006).

8. Pan, X. et al. *Mycolicibacterium aurantiacum* sp. nov. and *Mycolicibacterium xanthum* sp. nov., two novel actinobacteria isolated from mangrove sediments. *Int J Syst Evol Microbiol* **72**, 005595 (2022).

9. Tsukamura, M. Adansonian classification of mycobacteria. *J Gen Microbiol* **45**, 253-273 (1966).

10. Tsukamura, M., Van Der Meulen, H. J. & Grabow, W. O. K. Numerical taxonomy of rapidly growing, scotochromogenic mycobacteria of the *Mycobacterium parafortuitum* complex: *Mycobacterium austroafricanum* sp. nov. and *mycobacterium diernhoferi* sp. nov., nom. rev. *Int J Syst Bacteriol* **33**, 460-469 (1983).

11. Brown-Elliott, B. A. et al. *Mycobacterium neoaurum* and *Mycobacterium bacteremicum* sp. nov. as causes of mycobacteremia. *J Clin Microbiol* **48**, 4377-4385 (2010).

12. Cheng, Y. et al. *Mycolicibacterium baixiangningiae* sp. nov. and *Mycolicibacterium mengxianglii* sp. nov., two new rapidly growing mycobacterial species. *Int J Syst Evol Microbiol* **71**, 005019 (2021).

13. Schinsky, M. F. et al. Taxonomic variation in the *Mycobacterium fortuitum* third biovariant complex: description of *Mycobacterium boenickei* sp. nov., *Mycobacterium houstonense* sp. nov., *Mycobacterium neworleansense* sp. nov. and *Mycobacterium brisbanense* sp. nov. and recognition of *Mycobacterium porcinum* from human clinical isolates. *Int J Syst Evol Microbiol* **54**, 1653-1667 (2004).

14. Luquin, M. et al. *Mycobacterium brumae* sp. nov., a rapidly growing, nonphotochromogenic *mycobacterium*. *Int. J. Syst. Bacteriol* **43**, 405-413 (1993).

15. Soledad Jimenez, M. et al. *Mycobacterium canariasense* sp. nov. *Int J Syst Evol Microbiol* **54**, 1729-1734 (2004).

16. Shahraki, A. H. et al. *Mycobacterium celeriflavum* sp. nov., a rapidly growing scotochromogenic bacterium isolated from clinical specimens. *Int J Syst Evol Microbiol* **65**, 510-515 (2015).

17. Tsukamura, M. *Mycobacterium chitae*: a new species. *Jpn J Microbiol* **11**, 43-47 (1967).

18. Apajalahti, J. H. A., Karpanoja, P. & Salkinoja-Salonen, M. S. *Rhodococcus chlorophenolicus* sp. nov., a chlorophenol-mineralizing actinomycete. *Int. J. Syst. Bacteriol* **36**, 246-251 (1986).

19. Kirschner, P. et al. *Mycobacterium confluentis* sp. nov. *Int J Syst Bacteriol* **42**, 257-262 (1992).

20. Cooksey, R. C. et al. *Mycobacterium cosmeticum* sp. nov., a novel rapidly growing species isolated from a cosmetic infection and from a nail salon. *Int J Syst Evol Microbiol* **54**, 2385-2391 (2004).

21. Tortoli, E. et al. *Mycobacterium doricum* sp nov. *Int J Syst Evol Microbiol* **51**, 2007-2012 (2001).

22. Stanford, J. L. & Gunthorpe, W. J. A study of some fast-growing scotochromogenic mycobacteria including species descriptions of *Mycobacterium gilvum* (new species) and *Mycobacterium duvalii* (new species). *Br J Exp Pathol* **52**, 627-637 (1971).

23. Shojaei, H. et al. *Mycobacterium elephantis* sp. nov., a rapidly growing non-chromogenic *Mycobacterium* isolated from an elephant. *Int J Syst Evol Microbiol* **50**, 1817-1820 (2000).

24. Levy-Frebault, V. et al. *Mycobacterium fallax* sp. nov. *Int. J. Syst. Bacteriol* **33**, 336-343 (1983).

25. Chamoiseau, G. *M. farcinogenes* agent causal du farcin du boeuf en Afrique. *Ann. Microbiol. Inst. Pasteur* **124**, 215-222 (1973).

26. Bojalil, L. F., Cerbon, J. & Trujillo, A. Adansonian classification of mycobacteria. *J Gen Microbiol* **28**, 333-346 (1962).

27. Hormisch, D. et al. *Mycobacterium fluoranthenivorans* sp. nov., a fluoranthene and aflatoxin B1 degrading bacterium from contaminated soil of a former coal gas plant. *Syst Appl Microbiol* **27**, 653-660 (2004).

28. Da Costa Cruz, J. C. *Mycobacterium fortuitum* um novo bacillo acidoresistance pathogenico para o homen. *Acta Medica (Rio de Janeiro)* **1**, 297-301 (1938).

29. Willumsen, P. et al. *Mycobacterium frederiksbergense* sp. nov., a novel polycyclic aromatic hydrocarbon-degrading *Mycobacterium* species. *Int J Syst Evol Microbiol* **51**, 1715-1722 (2001).

30. Casal, M. & Calero, J. R. *Mycobacterium gadium* sp. nov. a new species of rapid-growing scotochromogenic mycobacteria. *Tubercle* **55**, 299-308 (1974).

31. Brown, B. A. et al. *Mycobacterium wolinskyi* sp. nov. and *Mycobacterium goodii* sp. nov., two new rapidly growing species related to *Mycobacterium smegmatis* and associated with human wound infections: a cooperative study from the International Working Group on Mycobacterial Taxonomy. *Int J Syst Bacteriol* **49**, 1493-1511 (1999).

32. Huang, R.-R. et al. Genomic molecular signatures determined characterization of *Mycolicibacterium gossypii* sp. nov., a fast-growing mycobacterial species isolated from cotton field soil. *Antonie Van Leeuwenhoek* **114**, 1735-1744 (2021).

33. Schroder, K. H. et al. *Mycobacterium hassiacum* sp. nov., a new rapidly growing thermophilic *mycobacterium*. *Int J Syst Bacteriol* **47**, 86-91 (1997).

34. Tran, P. M. & Dahl, J. L. *Mycobacterium sarraceniae* sp. nov. and *Mycobacterium helvum* sp. nov., isolated from the pitcher plant Sarracenia purpurea. *Int J Syst Evol Microbiol* **66**, 4480-4485 (2016).

35. Balcazar, J. L., Planas, M. & Pintado, J. *Mycobacterium hippocampi* sp. nov., a rapidly growing scotochromogenic species isolated from a seahorse with tail rot. *Curr Microbiol* **69**, 329-333 (2014).

36. Kleespies, M. et al. *Mycobacterium hodleri* sp. nov., a new member of the fast-growing mycobacteria capable of degrading polycyclic aromatic hydrocarbons. *Int J Syst Bacteriol* **46**, 683-687 (1996).

37. Richter, E. et al. *Mycobacterium holsaticum* sp. nov. *Int J Syst Evol Microbiol* **52**, 1991-1996 (2002).

38. Tortoli, E. et al. *Mycobacterium insubricum* sp. nov. *Int J Syst Evol Microbiol* **59**, 1518-1523 (2009).

39. Shojaei, H. et al. *Mycobacterium iranicum* sp. nov., a rapidly growing scotochromogenic species isolated from clinical specimens on three different continents. *Int J Syst Evol Microbiol* **63**, 1383-1389 (2013).

40. Gcebe, N. et al. *Mycobacterium komaniense* sp. nov., a rapidly growing non-tuberculous *Mycobacterium* species detected in South Africa. *Int J Syst Evol Microbiol* **68**, 1526-1532 (2018).

41. Kazda, J., and Muller, K. *Mycobacterium komossense* sp. nov. *Int. J. Syst. Bacteriol* **29**, 361-365 (1979).

42. Xiao, Y. et al. *Mycolicibacterium lacusdiani* sp. nov., an Attached Bacterium of Microcystis aeruginosa. *Front Microbiol* **13**, 861291 (2022).

43. Zhang, Y. et al. *Mycobacterium litorale* sp. nov., a rapidly growing *mycobacterium* from soil. *Int J Syst Evol Microbiol* **62**, 1204-1207 (2012).

44. Gomila, M. et al. *Mycobacterium llatzerense* sp. nov., a facultatively autotrophic, hydrogen-oxidizing bacterium isolated from haemodialysis water. *Int J Syst Evol Microbiol* **58**, 2769-2773 (2008).

45. Konjek, J. et al. *Mycobacterium lutetiense* sp. nov., *Mycobacterium montmartrense* sp. nov. and *Mycobacterium arcueilense* sp. nov., members of a novel group of non-pigmented rapidly growing mycobacteria recovered from a water distribution system. *Int J Syst Evol Microbiol* **66**, 3694-3702 (2016).

46. Kazda, J. et al. *Mycobacterium madagascariense* sp. nov. *Int J Syst Bacteriol* **42**, 524-528 (1992).

47. Domenech, P. et al. *Mycobacterium mageritense* sp. nov. *Int J Syst Bacteriol* **47**, 535-540 (1997).

48. Gcebe, N. et al. *Mycobacterium malmesburyense* sp. nov., a non-tuberculous species of the genus *Mycobacterium* revealed by multiple gene sequence characterization. *Int J Syst Evol Microbiol* **67**, 832-838 (2017).

49. Reischl, U. et al. *Mycobacterium monacense* sp. nov. *Int J Syst Evol Microbiol* **56**, 2575-2578 (2006).

50. Tsukamura, M., Yano, I. & Imaeda, T. *Mycobacterium moriokaense* sp. nov., a rapidly growing, nonphotochromogenic *Mycobacterium*. *Int. J. Syst. Bacteriol* **36**, 333-338 (1986).

51. Springer, B. et al. Phylogeny of the *Mycobacterium chelonae-*like organism based on partial sequencing of the 16S rRNA gene and proposal of *Mycobacterium mucogenicum* sp. nov. *Int J Syst Bacteriol* **45**, 262-267 (1995).

52. Tsukamura, M. *Mycobacterium neoaurum* Tsukamura sp. nov. *Med. Biol* **85**, 229-233 (1972).

53. Dahl, J. L., Gatlin Iii, W., Tran, P. M. & Sheik, C. S. *Mycolicibacterium nivoides* sp. nov isolated from a peat bog. *Int J Syst Evol Microbiol* **71**, 004438 (2021).

54. Shojaei, H. et al. *Mycobacterium novocastrense* sp. nov., a rapidly growing photochromogenic *Mycobacterium*. *Int J Syst Bacteriol* **47**, 1205-1207 (1997).

55. Tsukamura, M. *Mycobacterium parafortuitum*: a new species. *J Gen Microbiol* **42**, 7-12 (1966).

56. Kusunoki, S. & Ezaki, T. Proposal of *Mycobacterium peregrinum* sp. nov., nom. rev., and elevation of *Mycobacterium chelonae* subsp. *abscessus* (Kubica et al.) to species status: *Mycobacterium abscessus* comb. nov. *Int J Syst Bacteriol* **42**, 240-245 (1992).

57. Lehmann, K. B. et al. Atlas und Grundriss der Bakteriologie und Lehrbuch der speziellen Bakteriologishcen Diagnostik. *2. Aufl* **4**, 1-497 (1899).

58. Tsukamura, M., Nemoto, H. & Yugi, H. *Mycobacterium porcinum* sp. nov., a porcine pathogen. *Int. J. Syst. Bacteriol* **33**, 162-165 (1983).

59. Padgitt, P. J. & Moshier, S. E. *Mycobacterium poriferae* sp. nov., a scotochromogenic, rapidly growing species isolated from a marine sponge. *Int. J. Syst. Bacteriol* **37**, 186-191 (1987).

60. Trujillo, M. E. et al. *Mycobacterium psychrotolerans* sp. nov., isolated from pond water near a uranium mine. *Int J Syst Evol Microbiol* **54**, 1459-1463 (2004).

61. Tsukamura, M., Mizuno, S. & Toyama, H. *Mycobacterium pulveris* sp. nov., a nonphotochromatogenic *mycobacterium* with an intermediate growth rate. *Int J Syst Bacteriol* **33**, 811-815 (1983).

62. Derz, K. et al. *Mycobacterium pyrenivorans* sp. nov., a novel polycyclic-aromatic-hydrocarbon-degrading species. *Int J Syst Evol Microbiol* **54**, 2313-2317 (2004).

63. Chamoiseau, G. *M. farcinogenes* agent causal du farcin du bœuf en Afrique. *Annales de Microbiologie de l'Institut Pasteur (Paris)* **124**, 215-222 (1973).

64. Schinsky, M. F. et al. *Mycobacterium septicum* sp nov., a new rapidly growing species associated with catheter-related bacteraemia. *Int J Syst Evol Microbiol* **50**, 575-581 (2000).

65. Lamy, B. et al. *Mycobacterium setense* sp. nov., a *Mycobacterium fortuitum*-group organism isolated from a patient with soft tissue infection and osteitis. *Int J Syst Evol Microbiol* **58**, 486-490 (2008).

66. Kazda, J. *Mycobacterium sphagni* sp. nov. *Int. J. Syst. Bacteriol* **30**, 77-81 (1980).

67. Nouioui, I. et al. *Mycolicibacterium stellerae* sp. nov., a rapidly growing scotochromogenic strain isolated from Stellera chamaejasme. *Int J Syst Evol Microbiol* **69**, 3465-3471 (2019).

68. Tortoli, E. et al. *Mycobacterium tusciae* sp. nov. *Int J Syst Bacteriol* **49**, 1839-1844 (1999).

69. Bonicke, R. & Juhasz, S. T. Beschreibung der neuen Species *Mycobacterium vaccae* n. sp. *Zentralbl Bakteriol Orig* **192**, 133-135 (1964).

70. Cortes-Albayay, C. et al. Comparative Genomic Study of Vinyl Chloride Cluster and Description of Novel Species, *Mycolicibacterium vinylchloridicum* sp. nov. *Front Microbiol* **12**, 767895 (2021).

71. Hashemi Shahraki, A. et al. *Mycobacterium aquaticum* sp. nov., a rapidly growing species isolated from haemodialysis water. *Int J Syst Evol Microbiol* **67**, 3279-3282 (2017).

72. Adékambi, T., Raoult, D. & Drancourt, M. *Mycobacterium barrassiae* sp. nov., a *Mycobacterium moriokaense* group species associated with chronic pneumonia. *J Clin Microbiol* **44**, 3493-3498 (2006).

73. He, Y. et al. Whole-Genome Sequence of the 1,4-Dioxane-Degrading Bacterium *Mycobacterium dioxanotrophicus* PH-06. *Genome Announc* **5**, e00625-00617 (2017).

74. Gharbi, R. et al. Phenotypic and genomic hallmarks of a novel, potentially pathogenic rapidly growing *Mycobacterium* species related to the *Mycobacterium fortuitum* complex. *Sci Rep* **11**, 13011 (2021).

75. Tsukamura, M. et al. Bacteriological studies on atypical Mycobacteria isolated in Japan. 3. A comparison between pathogenic scotochromogens and soil scotochromogens--origin of pathogenic scotochromogens. *Kekkaku* **42**, 15-21 (1967).

76. Paniz-Mondolfi, A. E. et al. *Mycobacterium grossiae* sp. nov., a rapidly growing, scotochromogenic species isolated from human clinical respiratory and blood culture specimens. *Int J Syst Evol Microbiol* **67**, 4345-4351 (2017).

77. Hong, T. et al. Characterization of a novel rapidly growing *Mycobacterium* species associated with sepsis. *J Clin Microbiol* **41**, 5650-5653 (2003).

78. Nouioui, I. et al. Description of a novel species of fast growing *mycobacterium*: *Mycobacterium kyogaense* sp. nov., a scotochromogenic strain received as *Mycobacterium vaccae*. *Int J Syst Evol Microbiol* **68**, 3726-3734 (2018).

79. Nouioui, I. et al. Two novel species of rapidly growing mycobacteria: *Mycobacterium lehmannii* sp. nov. and *Mycobacterium neumannii* sp. nov. *Int J Syst Evol Microbiol* **67**, 4948-4955 (2017).

80. Turenne, C. Y. et al. Soft tissue infection caused by a novel pigmented, rapidly growing *Mycobacterium* species. *J Clin Microbiol* **41**, 2779-2782 (2003).

81. Phelippeau, M. et al. "*Mycobacterium massilipolynesiensis*" sp nov., a rapidly-growing *mycobacterium* of medical interest related to *Mycobacterium phlei*. *Sci Rep* **7**, 40443 (2017).

82. Nouioui, I. et al. Formal description of *Mycobacterium neglectum* sp. nov. and *Mycobacterium palauense* sp. nov., rapidly growing actinobacteria. *Antonie Van Leeuwenhoek* **111**, 1209-1223 (2018).

83. Musser, E. et al. Characterization of *Mycobacterium salfingeri* sp. nov.: A novel nontuberculous mycobacteria isolated from a human wound infection. *Front Microbiol* **13**, 992610 (2022).

84. Fogelson, S. B. et al. *Mycobacterium syngnathidarum* sp. nov., a rapidly growing *mycobacterium* identified in syngnathid fish. *Int J Syst Evol Microbiol* **68**, 3696-3700 (2018).

85. Gupta, R. S., Lo, B. & Son, J. Phylogenomics and Comparative Genomic Studies Robustly Support Division of the Genus *Mycobacterium* into an Emended Genus *Mycobacterium* and Four Novel Genera. *Front Microbiol* **9**, 67 (2018).

86. Oren, A. & Garrity, G. List of new names and new combinations previously effectively, but not validly, published. *Int J Syst Evol Microbiol* **68**, 1411-1417 (2018).

87. Tortoli, E. et al. Same meat, different gravy: ignore the new names of mycobacteria. *Eur Respir J* **54**, 1900795 (2019).

88. Meehan, C. J. et al. Reconstituting the genus *Mycobacterium*. *Int J Syst Evol Microbiol* **71**, 004922 (2021).

89. Tortoli, E. On the valid publication of names of mycobacteria. *Eur Respir J* **54**, 1901623 (2019).

90. Asmar, S. et al. Draft Genome Sequence of *Mycobacterium acapulcensis* Strain CSURP1424. *Genome Announc* **4**, e00836-00816 (2016).

91. Tortoli, E. et al. Genome-based taxonomic revision detects a number of synonymous taxa in the genus *Mycobacterium*. *Infect Genet Evol* **75**, 103983 (2019).

92. Gupta, R. S. Commentary: Genome-Based Taxonomic Classification of the Phylum *Actinobacteria*. *Front Microbiol* **10**, 206 (2019).

93. Turenne, C. Y. Nontuberculous mycobacteria: Insights on taxonomy and evolution. *Infect Genet Evol* **72**, 159-168 (2019).
